# A catalog of numerical centrosome defects in epithelial ovarian cancers

**DOI:** 10.15252/emmm.202215670

**Published:** 2022-09-07

**Authors:** Jean‐Philippe Morretton, Anthony Simon, Aurélie Herbette, Jorge Barbazan, Carlos Pérez‐González, Camille Cosson, Bassirou Mboup, Aurélien Latouche, Tatiana Popova, Yann Kieffer, Anne‐Sophie Macé, Pierre Gestraud, Guillaume Bataillon, Véronique Becette, Didier Meseure, André Nicolas, Odette Mariani, Anne Vincent‐Salomon, Marc‐Henri Stern, Fatima Mechta‐Grigoriou, Sergio Roman Roman, Danijela Matic Vignjevic, Roman Rouzier, Xavier Sastre‐Garau, Oumou Goundiam, Renata Basto

**Affiliations:** ^1^ Biology of Centrosomes and Genetic Instability, Institut Curie PSL Research University, CNRS UMR 144 Paris France; ^2^ Department of Translational Research, Institut Curie PSL University Paris Cedex 05 France; ^3^ Migration and Invasion Laboratory, Institut Curie PSL Research University, CNRS UMR 144 Paris France; ^4^ Statistical Methods for Precision Medicine INSERM U900, Institut Curie Saint‐Cloud France; ^5^ DNA Repair & Uveal Melanoma (D.R.U.M.), INSERM U830, Institut Curie PSL Research University Paris Cedex 05 France; ^6^ Stress and Cancer Laboratory, INSERM U830, Institut Curie, Team Ligue Nationale Contre le Cancer PSL Research University Paris France; ^7^ Cell and Tissue Imaging Facility (PICT‐IBiSA), Institut Curie PSL Research University, Centre National de la Recherche Scientifique Paris France; ^8^ Bioinformatics and Computational Systems Biology of Cancer, Mines Paristech, INSERM U900, Institut Curie PSL University Paris Cedex 05 France; ^9^ Department of Pathology Institut Curie Paris Cedex 05 France; ^10^ Biological Resource Center, Department of Pathology, Institut Curie PSL Research University Paris France; ^11^ Department of Surgery Institut Curie Saint‐Cloud France; ^12^ UFR Simone Veil – Santé Université Versailles Saint Quentin, Université Paris Saclay Montigny le Bretonneux France; ^13^ Present address: Laboratory of Pathology Intercommunal Hospital Center of Creteil Creteil Cedex France

**Keywords:** centrosomes, ovarian cancers, centrosome number alterations, Cancer, Urogenital System

## Abstract

Centrosome amplification, the presence of more than two centrosomes in a cell is a common feature of most human cancer cell lines. However, little is known about centrosome numbers in human cancers and whether amplification or other numerical aberrations are frequently present. To address this question, we have analyzed a large cohort of primary human epithelial ovarian cancers (EOCs) from 100 patients. We found that rigorous quantitation of centrosome number in tumor samples was extremely challenging due to tumor heterogeneity and extensive tissue disorganization. Interestingly, even if centrosome clusters could be identified, the incidence of centrosome amplification was not comparable to what has been described in cultured cancer cells. Surprisingly, centrosome loss events where a few or many nuclei were not associated with centrosomes were clearly noticed and overall more frequent than centrosome amplification. Our findings highlight the difficulty of characterizing centrosome numbers in human tumors, while revealing a novel paradigm of centrosome number defects in EOCs.

## Graphical abstract

Characterization of the centrosome number in epithelial ovarian cancers (EOCs) is challenging because these tumors are extremely disorganized and heterogeneous in respect to centrosome number.

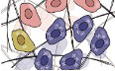

**The paper explained**

**Problem**

Epithelial ovarian cancers (EOCs) are among the most deadly cancers in women worldwide. EOCs are considered to be hard to treat due to late diagnosis and poor patient stratification. It is currently accepted that new parameters should be defined to attempt to better characterize these tumors.

**Results**

The centrosome is the major microtubule‐organizing center of animal cells with important functions in mitotic spindle establishment, cell cycle progression, polarity, and cell migration. Using state‐of‐the‐art microscopy, we imaged and analyzed centrosome numbers in a large cohort of 100 EOCs and compared it with healthy tissues. We report that quantification of centrosome numbers is very challenging in tumors that are extremely disorganized such as EOCs. Using different centrosome markers, we found that EOCs are highly heterogeneous in terms of centrosome number defects. We show that many EOC cells lack centrosomes and only a small population contains amplified centrosomes.

**Impact**

This study shows that centrosome amplification is less common in EOCs than in cultured cancer cells. It reveals a novel and frequent type of numerical aberration, centrosome loss, that deserves further attention.

## Introduction

The centrosome is the main microtubule‐organizing center of animal cells. Each centrosome is composed of two centrioles surrounded by pericentriolar material (PCM), which is the site of microtubule nucleation. The centrosome facilitates the accuracy of chromosome segregation during mitosis and influences cell polarity and migration (Bettencourt‐Dias & Glover, 2007; Bornens, 2012). The presence of more than two centrosomes in a cell, centrosome amplification, is associated with tumorigenesis. T. Boveri proposed for the first time, more than 100 years ago, a link between extra centrosomes, multipolar divisions, and aneuploidy (Boveri, 2008). When induced by manipulating the centrosome duplication machinery, centrosome amplification is sufficient to drive tumor formation *in vivo* in various tissues in different animal models (Basto *et al*, 2008; Coelho *et al*, 2015; Serçin *et al*, 2016; Levine *et al*, 2017).

Although centrosome amplification is generally associated with abnormal cell division and so aneuploidy (Boveri, 2008; Ganem *et al*, 2009; Silkworth *et al*, 2009; Sabino 2015; Serçin *et al*, 2016; Levine *et al*, 2017; Raff & Basto, 2017), centrosome amplification can also impact cellular homeostasis in alternative ways leading to cell invasion (Godinho *et al*, 2014; Arnandis *et al*, 2018). Additionally, noncell autonomous detachment of mitotic tumor cells is described in organoids containing increased levels of Ninein‐like protein, which induces centrosome structural defects (Casenghi *et al*, 2003; Schnerch & Nigg, 2016; Ganier *et al*, 2018).

Epithelial ovarian cancers (EOCs) are the most lethal gynecologic malignancies (Berns & Bowtell, 2012). The high mortality rate is a result of late diagnosis and limited therapeutic options despite the use of new drugs, such as inhibitors of angiogenesis or DNA repair pathways (Konstantinopoulos *et al*, 2015; Pujade‐Lauraine *et al*, 2017). The histological classification includes mainly serous, endometrioid, mucinous, and clear cell carcinomas. The most common EOCs subtype is high‐grade serous (HGSOC), which presents a worse overall prognosis (Ramalingam, 2016).

Even though numerical centrosome defects are described in different cultured cancer cell types (Marteil *et al*, 2018), few studies have described centrosome number alterations in tumors *in situ* (Zyss & Gergely, 2009; Goundiam & Basto, 2021). Here, we used a large EOCs cohort composed of 100 naive tumors comprising 88 HGSOCs.

## Results

### Characterization of centrosome defects in human EOC tissues

To analyze centrosomes in human EOCs, we obtained frozen tissue sections from the pathology department of Institut Curie. These were categorized as healthy tissues (corresponding to healthy ovaries from prophylactic oophorectomy or hysterectomy) or tumor tissues, including a mix of serous (90%), endometrioid (3%), mucinous (4%), and clear cell carcinoma (3%) (Materials and Methods and Table EV1). All tumors were treatment‐naïve, obtained after surgery without previous neo‐adjuvant chemotherapy.

**Figure 1:**
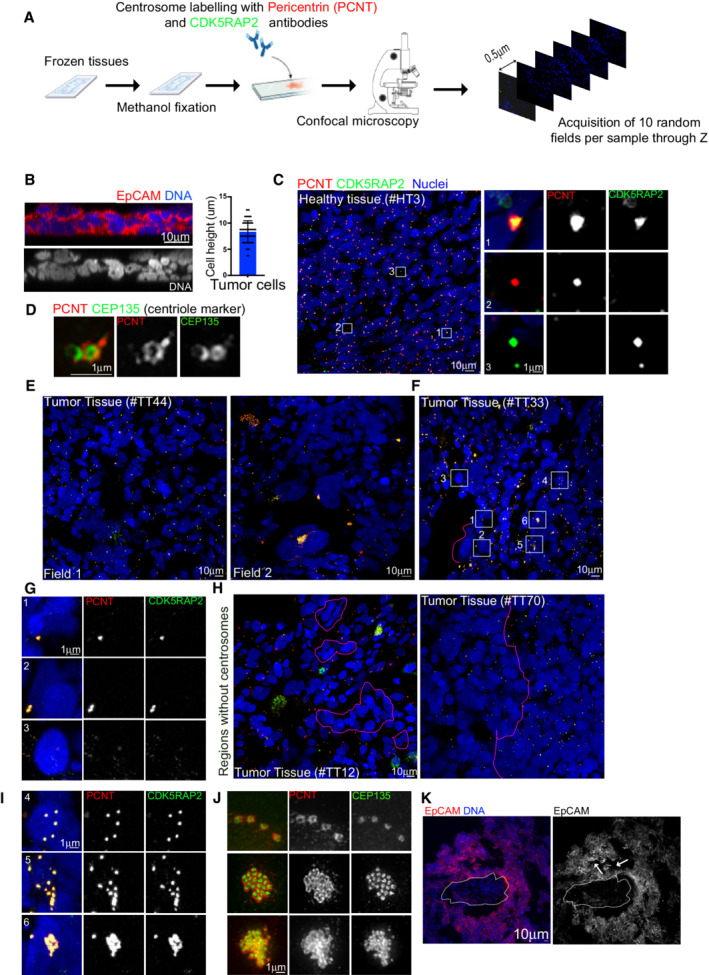
Characterization of centrosome numbers in healthy ovarian tissues ASchematic diagram of the workflow used to analyze ovarian tissue sections.BLeft, orthogonal view of a 20 μm tumor section labeled with antibodies against EpCAM (in red) and DNA in blue. Right, dot plot bar representing cell heights in cells from tumor sections, *n* = 60 cells from 1 tumor. Mean ± SD.COn the left, images of low magnification views of healthy tissues labeled with antibodies against pericentrin (PCNT) and CDK5RAP2, shown in red and green, respectively. DNA in blue. The white dashed squares represent the regions shown in higher magnifications on the right. One centrosome was considered as such when PCNT and CDK5RAP2 signals co‐localized (Inset 1). Lack of co‐localization (Insets 2 and 3) was noticed and discarded during quantification.DSuper‐resolution microscopy of healthy tissues labeled for the centriole marker CEP135 (in green) and PCNT (in red).E, FRepresentative images of two different fields of the same tumor (E) and of a tumor section to highlight different centrosome phenotypes, labeled as described in (C). The pink dashed line highlights an area of cells without centrosomes. The white‐dashed squares represent the regions shown in higher magnifications in insets in (G and I) and the pink dashed line surrounds nuclei without centrosomes.GInsets from (F).HExamples of tumor sections showing small (left) and large (right) regions with nuclei without centrosomes.IInsets from (F) showing different types of centrosome amplification events.JSuper‐resolution microscopy of tumor tissues labeled as in (C).KMaximum Z‐projection of a tumor section labeled with EpCAM antibodies (Red) and DNA in blue. The dashed line shows the absence of EpCAM staining, arrows show a disorganized region.

Tissues were labeled for Pericentrin (PCNT) and CDK5RAP2 using commercially available antibodies, which enable us to use large quantities of these reagents, required in this type of approach. PCNT and CDK5RAP2 are PCM components, and through their co‐localization we can unambiguously identify centrosomes as defined in previous studies (Basto *et al*, 2008; Serçin *et al*, 2016; Gambarotto *et al*, 2019). Using confocal microscopy, we obtained optical Z sections spanning a total of 20 μm from 10 random fields in the entire tissue (Fig 1A). We analyzed 20 μm sections as the quantification of cell heights within the tumor tissues revealed an average height of 8.33 ± 2.1 μm (Fig 1B). We reasoned that the 20‐μm stack would allow us to safely identify all centrosomes of a given cell.

Analysis of healthy tissues permitted us to identify centrosomes (Fig 1C, inset 1). We also noticed the presence of structures that only contained one of the two centrosome markers (Fig 1C, insets 2 and 3). These were not considered centrosomes. To further characterize and confirm the centrosomal configurations described above, we used 3D structural illumination microscopy (3D‐SIM) of ovarian tissues labeled with the centriolar marker‐CEP135 and PCNT, allowing higher resolution for both centrioles and PCM (Fig 1D). We found that in healthy tissues, each centrosome contained two centrioles and as expected (Conduit *et al*, 2015), PCNT surrounded one of the two centrioles, presumably the mother centriole.

Analysis of tumor tissues revealed the presence of highly heterogeneous phenotypes in respect to centrosome numbers and overall aspect of the tissue (Fig 1E, fields #1 and #2 from the same tumor). This supports the requirement for the acquisition of multiple fields for each tumor. In tumor sections, one or two centrosomes were readily noticed (Fig 1F and G, insets 1–2). Surprisingly, however, in other nuclei, we could not detect centrosomes or even any signal from individual centrosome proteins (Fig 1G and H, inset 3). The lack of centrosomes was easily identified in groups of nuclei of different size (Fig 1H‐left), but also in considerable regions spanning large fractions of the tumor section (Fig 1H‐right).

Considering centrosome amplification, in certain cells extra centrosomes could be seen as isolated structures spread away from each other (Fig 1I, inset 4), as isolated centrosomes. In other cells, they were clustered together—clustered centrosomes (Fig 1I, inset 5). Interestingly, we also observed a configuration where extra centrosomes were tightly clustered in a single structure—super‐clusters (Fig 1I, inset 6). SIM analysis of these tumors, with the markers described above, demonstrated the unusual extra centrosome morphologies (Fig 1J). Importantly, centrosome amplification or lack of centrosomes were not detected in healthy tissues. Still considering the tumors, we failed to obtain a reliable and reproducible signal using membrane markers in tumor tissues (Fig 1K). In certain regions, the membrane showed invaginations and appeared very deformed, hampering the correlation of a given centrosome to a given nucleus.

Altogether, the methodology employed to analyze 100 ovarian tumors and the comparison with healthy ovarian tissues revealed highly disorganized tumor tissues and the unexpected presence of cells without centrosomes.

### EOCs show heterogeneous centrosome number alterations

**Figure 2:**
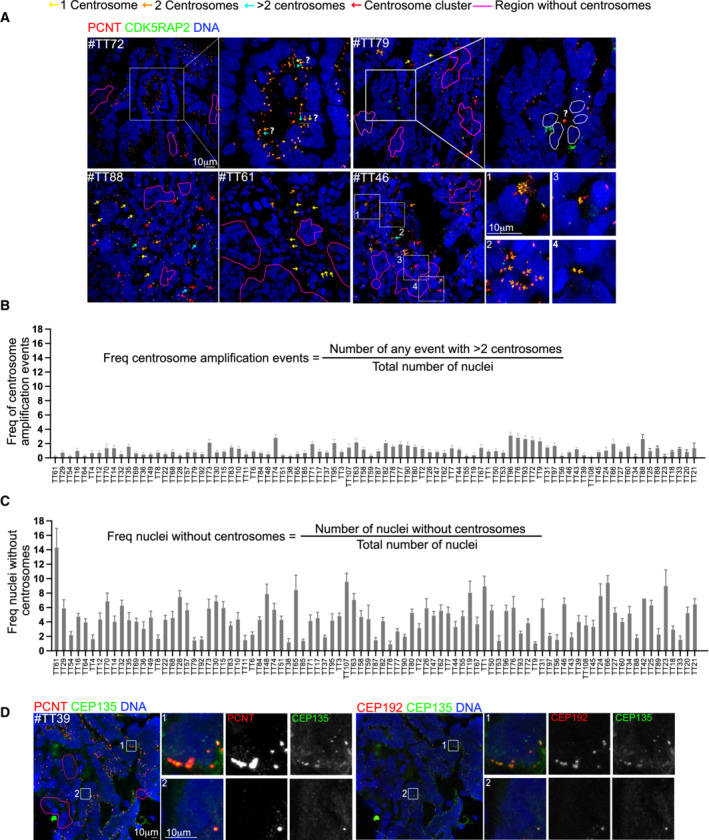
EOCs display highly heterogeneous centrosome number alterations AImages of EOC tumors labeled with antibodies against PCNT (red) and CDKRAP2 (green). Nuclei are shown in blue. The colored arrows reflect different centrosome number. The list of centrosome behaviors annotated it is not exhaustive to allow comprehension of the events. #TT72 shows a cyst‐like arrangement of EOC nuclei where it is impossible to correspond centrosomes to individual nuclei. The white bars with arrows of different colors highlight possible interpretations of the same condition. In #TT79, regions without centrosomes can be identified. In the inset, a single centrosome localized in the lumen can be identified and it is not possible to know to which nucleus (white dashed lines) it corresponds to. #TT88 presents only very small clusters of centrosomes, few nuclei without centrosomes and many conditions with two centrosomes. In #TT61, regions without centrosomes can be distinguished, in addition to nuclei associated with single centrosomes. In #TT46, clusters containing large (inset 1) or small clusters (inset 3) can be identified. In cyst‐like arrangements several centrosomes can be identified (inset 2), suggesting that all cells have at least one centrosome or, in contrast just one centrosome (inset 4), illustrating lack of centrosomes.B, CPlots showing the frequency of centrosome amplification events (B) and the frequency of nuclei without centrosomes (C) in tumor tissues. Note that in B and C, the order of the tumors is conserved between the two plots to allow for comparison. For each tumor, 10 random fields were chosen and analyzed, with an average of 5,248 nuclei counted per tumor. Bars represent the mean ± SD.DTumor sections labeled with CEP135 (green) and PCNT (red) on the left and CEP135 (green) and CEP192 (red) on the right. The white squares represent the regions shown in higher magnifications on the right.

We next analyzed tumor sections focusing on regions corresponding exclusively to the tumor, excluding the stroma that surrounds the tumor. Tumor tissues appeared extremely disorganized when compared to healthy tissues, and frequently, it was difficult to ascertain the number of centrosomes per cell. Indeed, in certain regions, it was difficult to interpret if a particular set of centrosomes corresponded to a single nucleus or to several nuclei (Fig 2A, top left panel #TT72). Moreover, centrosomes were not easily identified as being associated with a given nucleus (Fig 2A top right panel #TT79).

Analysis of tumor sections revealed many different scenarios including a variability in the presence and absence of centrosome amplification and its extent, the number of nuclei without centrosomes and even in the number of nuclei with one or with two centrosomes (Fig 2A). For example, in certain sections, we could clearly identify all possible configurations: one, two, and three centrosomes and small centrosome clusters in addition to regions without centrosomes (Fig 2A, bottom left #TT88). In other sections, nuclei with centrosome amplification were not noticed, while regions lacking centrosomes were frequent (Fig 2A, bottom middle #TT61). Additionally, within the same tumor section centrosome clusters could contain different number of centrosome (Fig 2A, insets 1 and 3 #TT46) with certain clusters containing just a few, while others contain many centrosomes. Interestingly, nuclei were commonly arranged in cyst‐like arrangements with obvious differences in centrosome numbers (Fig 2A, insets 2 and 4 #TT46).

To ascertain the extent of centrosome number alterations in our tumor cohort, we quantified the frequency of centrosome amplification and loss (Fig 2B and C). This analysis revealed that the lack of centrosomes was more frequent than centrosome amplification as the former was present in all tumors, which was not the case of the latter, at least as obvious centrosome amplification events as shown in Fig 1E. Considering the frequency of centrosome amplification, it only reached a maximum of ~3.2% (Fig 2B). Interestingly, the number of centrosomes in events of centrosome amplification was very variable. For example, in #TT44 (Fig 1D, right panel), six large clusters of extra centrosomes could be easily distinguished. Some of these contained several dozens of centrosomes in total, while in other events clusters were of medium size #TT46 (Fig 2A inset 1) or quite small #TT46, (Fig 2A inset 3). The frequency of nuclei without centrosomes was also extremely variable (Fig 2C) and it was very difficult to evaluate if certain nuclei lacked centrosomes. Regions like the one depicted in (Fig 2A‐ #TT79) were excluded from the analysis as it is difficult to unambiguously identify which nucleus was not associated with centrosomes. It is thus possible that the frequency of nuclei without centrosomes is an underestimation.

Overall, these results show that subcellular characterization of organelles such as centrosomes, in highly heterogeneous tumor populations like the ones found in ovarian cancers is extremely complex.

A possible caveat of our experimental procedure was the use of PCM markers to quantify centrosome numbers. In light of this scenario, a likely explanation for the results described above, reporting low centrosome amplification levels, or even cells and regions lacking centrosomes, was that these cells contain centrioles that do not recruit the two PCM markers analyzed. This seems rather unlikely as even small centrosomes (Fig 1I, insets 4 or 5) were noticed through the co‐localization of PCNT and CDK5RAP2. Nevertheless, to analyze centrosome numbers in an alternative way, we used a combination of antibodies recognizing CEP135, CEP192, and PCNT. CEP135 interacts with the cartwheel SAS‐6 component, and it is recruited to the parental centriole (Kleylein‐Sohn *et al*, 2007; Sonnen *et al*, 2012), while CEP192 is one of the two scaffold proteins essential to recruit PLK4 (Hatch *et al*, 2010; Sonnen *et al*, 2013; Park *et al*, 2014). As before, we analyzed 20 μm sections of 23 tumors within our cohort and count centrosomes through the colocalization of these markers. As before, we could identify, within the same tumor and tumor section, nuclei with one or two centrosomes, nuclei associated with centrosome clusters, and nuclei without centrosomes (Fig 2D). It is important to mention that throughout the analysis, we noticed that in the large majority of centrosomes, PCNT displayed a signal very similar to any of the centriole markers (Fig 2D).

In conclusion, in EOCs inter‐ and intra‐tumor heterogeneity can be observed in terms of centrosome numbers. Surprisingly, only a small population of tumor cells displays extra centrosomes and many nuclei lack centrosomes, which is unexpected in tumors of epithelial origin.

### Ovarian cancer spheroids with low centrosome number do not show increased invasion or migration capacity

**Figure EV1:**
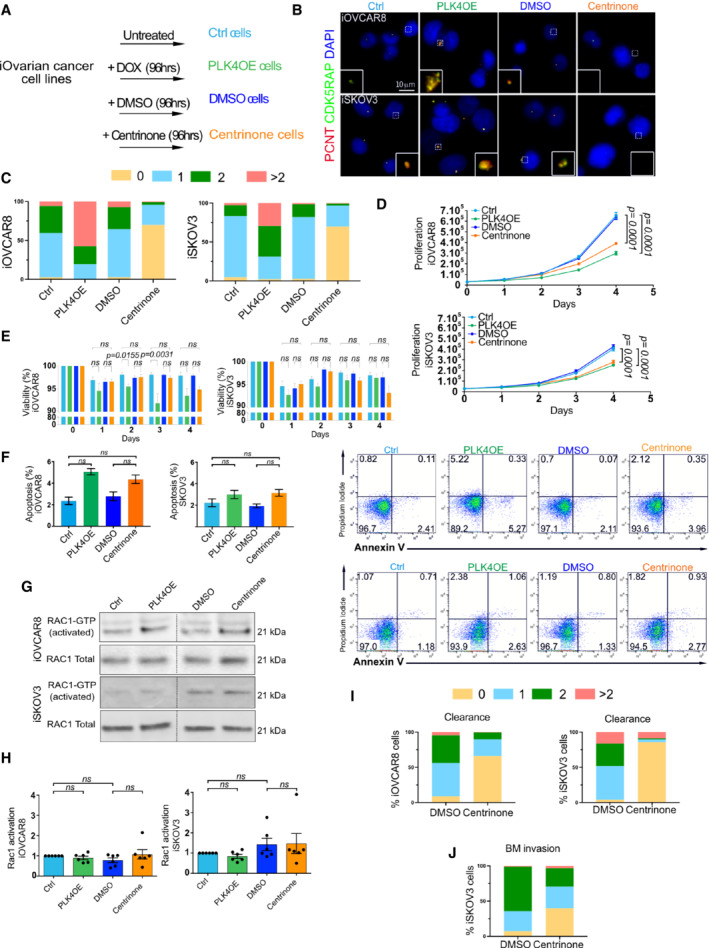
Characterization of ovarian cancer stable cell lines after gain or loss of centrosomes AScheme of the different experimental conditions and nomenclature used.BRepresentative images of iOVCAR8 (top) and iSKOV3 (bottom) after the indicated treatments labeled with antibodies against PCNT and CDK5RAP2 (red and green respectively, DNA in blue). The white dashed squares represent the regions shown in higher magnification on the bottom.CGraph bars representing the percentage of cells according to centrosome number after the different treatments of iOVCAR8 (left) and iSKOV3 (right), at least 80 cells analyzed by condition.DGraphs representing the proliferation of iOVCAR8 and iSKOV3 cells after each treatment for 4 days. From three replicates in three independent experiments, statistical significances assessed with two‐way ANOVA, *n* = 6 independent experiments. Mean ± SD.EGraph bars representing the viability of iOVCAR8 and iSKOV3 cells after the designated treatment according to indicated timing in days. From three replicates in three independent experiments, statistical significance was assessed with one‐way ANOVA, *n* = 6 independent experiments. Mean ± SD.FOn the left, graph bars represent the percentage of apoptotic cells iOVCAR8 and iSKOV3 after each indicated treatment. Statistical significances were assessed with the Wilcoxon test, *n* = 6 independent experiments (H). Mean ± SD. On the right, representative FACS plots showing Annexin V^+^ (*x*‐axis) and PI^+^ (*y*‐axis) cells, for iOVCAR8 (top) and iSKOV3 (bottom) cells after the indicated treatments. For all experiments, *n* = 3 independent experiments.G, HWestern blot and graphs bars representing the quantifications of active RAC‐1 in iOVCAR8 and iSKOV3 cell lines after the indicated treatments. Statistical significances were assessed with one‐way ANOVA, *n* = 6 independent experiments. Mean ± SD.I, JDot plot graphs showing the percentage of cells in each category according to centrosome number corresponding to the experiments of mesothelial cell clearance (I) and BM invasion assays (J). At least 80 cells were analyzed per condition. Source data are available online for this figure.

The high frequency of tumors containing cells without centrosomes prompted us to explore if abnormal centrosome numbers can influence ovarian cancer cell behavior. We performed these experiments *in vitro*, in an isogenic background and using ovarian cancer cell lines. We generated inducible‐(i) OVCAR8‐PLK4 and SKOV3‐PLK4 stable cell lines, where the expression of PLK4, the master centriole duplication, can be modulated. To increase centrosome numbers, PLK4 over‐expression (PLK4OE) can be induced using doxycycline (Dox) (Holland *et al*, 2012). To decrease centrosome number, we used centrinone, a PLK4 inhibitor (Wong *et al*, 2015). These cells will be referred to as centrinone cells. Treatment of either cell line with Dox or centrinone effectively impacted centrosome numbers (Fig EV1A–C). Although proliferation was decreased in Dox and centrinone‐treated cells, these cells still proliferated (Fig EV1D and E), and apoptosis was only mildly increased (Fig EV1F). OVCAR8 and SKOV3 are EOCs cell lines with mutations in p53, explaining the continued proliferation in response to centrosome number alterations, in contrast to diploid untransformed cell lines (Holland *et al*, 2012; Lambrus *et al*, 2015; Wong *et al*, 2015).

In MCF10A 3D cultures, centrosome amplification results in increased levels of an activated form of the small GTPase‐RAC1 (Godinho *et al*, 2014). In EOC cell lines, however, we did not observe any significant difference in the levels of activated RAC1 after Dox or centrinone treatments (Fig EV1G and H).

**Figure 3:**
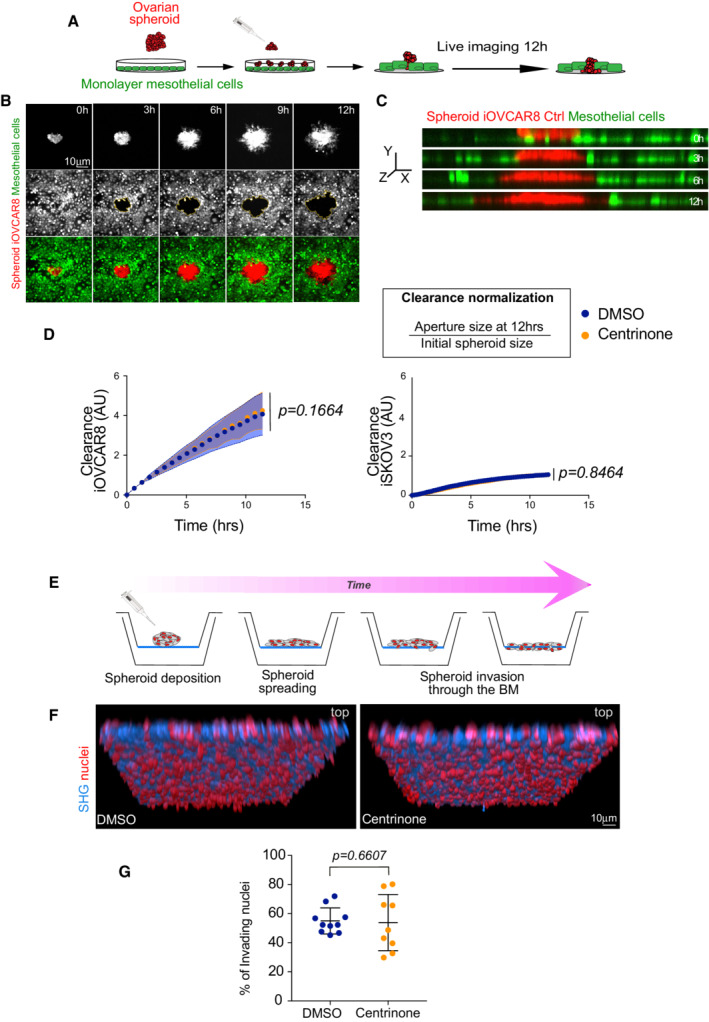
Ovarian cancer spheroids with low centrosome number do not show increased invasion or migration capacity ASchematic diagram of the experimental setup used in mesothelial cell clearance assays.BStills of a time‐lapse movie of Ctrl spheroids. Time is shown in hours (h).Cz‐ view of Ctrl cells as shown in (B). Note the red‐colored cancer cells at the beginning of the movie on top of the mesothelial layer (green) while at later time points, they have cleared through the mesothelial cells.DGraph bars of the normalized clearance in A.U. of iOVCAR8 (left) and iSKOV3 (right) spheroids after the indicated treatments. The two cell lines show different clearance capacity. For each cell line, three independent experiments were performed. For iOVCAR8, we analyzed 47 spheroids in DMSO and 56 spheroids in centrinone. For iSKOV3 we analyzed 56 spheroids in DMSO and 48 spheroids in centrinone. Dots represent the mean and the shadow ± SEM. Statistical significance was assessed with an ANCOVA test. Dots represent the mean and the shadow ± SEM. Statistical significance was assessed with an ANOVA test.ESchematic diagram of the experimental setup used in BM invasion assays.FRepresentative images of DMSO (left) and centrinone (right) spheroids. Mean ± SD. Red nuclei represent false colored invading nuclei.GDot plot showing the quantification of the number of nuclei detected on the bottom side of the BM. Three or four positions from three BM inserts were analyzed in each condition. Mean ± SD. Statistical significance was assessed with the Mann–Whitney test. For all the experiments, the centrosome number was verified in parallel to confirm the decreased centrosome conditions compared to DMSO.

Epithelial ovarian cancers undergo a particular mode of dissemination. Tumor cells detach from the primary tumor site, adhere to and migrate through the mesothelial cell layer that encloses peritoneal organs (Kipps *et al*, 2013), resulting in peritoneal metastasis (Iwanicki *et al*, 2011; Barbolina, 2018). Testing cell clearance of EOC cells using 3D spheroids plated on top of mesothelial cells (Figs 3A and EV1I) revealed by time‐lapse imaging analysis that decreased centrosome numbers did not influence mesothelial cell clearance when compared to controls (Fig 3B–D).

Next, we tested basement membrane (BM) invasion. We used decellularized mouse mesentery as an *ex‐vivo* model, that replicates the complex BM architecture located beneath the mesothelium (Schoumacher *et al*, 2013; Glentis *et al*, 2018; Fig 3E). Cancer cell spheroids were plated on mesenteries and after 7 days, we quantified invasion by counting the number of cells on the other side of the mesentery. We found that spheroids with decreased centrosome numbers have the same invasion capacity as control spheroids (Figs 3F and G, and EV1J).

Overall, our results show that centrosome loss does not impact migration or invasion in the ovarian cell models used in this study.

## Discussion

The analysis of a large cohort of EOCs identified centrosome loss as a characteristic of these tumors. Even if cells with extra centrosomes could be easily identified, their frequency was low in the large majority of the EOCs examined. The presence of cells without centrosomes has recently been described in human prostate tumors (Wang *et al*, 2020). Together, these two studies raise the novel possibility that at least in prostate and in ovarian cancers, centrosome loss is a frequent centrosome numerical aberration.

Healthy ovarian tissues contain one centrosome per nucleus. Since in all tumors, nuclei without centrosomes were detected, it is possible that during the malignant process, centrosomes do not duplicate or are mis‐segregated. Future work will be needed to elucidate the mechanisms responsible for centrosome loss in EOCs.

Epithelial ovarian cancers, and in particular HGSOCs, are highly aggressive and invasive. Patients often present tumor masses in peritoneal tissues due to cancer cell dissemination through ascites fluid (Lengyel, 2010). Our data suggest that centrosome loss is not translated into increased mesothelial clearance capacity or invasion through the BM, at least in the experimental conditions described here. These findings suggest that yet unidentified properties of low centrosome numbers in EOC cells may contribute to tumor dissemination.

Our work paves the way for the analyses of a high number of naïve human cancers to characterize the centrosome status. It will be essential to perform this type of study in other cancers to identify the frequencies of centrosome amplification and centrosome loss.

## Materials and Methods

### Experimental model and subject details

#### Ovarian cancer cohort

All 100 ovarian cancer samples included in this study were obtained from patients treated at the Institut Curie Hospital for epithelial ovarian cancer. Clinical data, including FIGO staging, were prospectively registered and summarized in Table EV1. After the pathology review of cryosections, frozen tissues were used for DNA, RNA, and proteins extractions and subsequent analysis. All samples were taken before chemotherapy administration and obtained from the Biological Resource Center (BRC) of Institut Curie (certification number: 2009/33837.4; AFNOR NF S 96 900). Normal ovarian tissues were obtained from hysterectomy or prophylactic oophorosalpingectomy.

According to French regulations, patients were informed of the studies performed on tissue specimens and did not express opposition. All analyses were approved by the National Commission for Data Processing and Liberties (No approval: 1487390), as well as the Institutional Review Board and Ethics committee of the Institut Curie. Experiments using human samples conformed to the principles set out in the WMA Declaration of Helsinki and the Department of Health and Human Services Belmont Report.

#### Cell culture

SKOV3 (ATCC^®^ #HTB‐77) cell lines used in this study were purchased from ATCC (LGC Promochem Sarl), OVCAR8 cells were obtained from the laboratory of F. Mechta‐Grigoriou. Ovarian cancer cell lines were cultured in DMEM/F12 media (Thermo Fisher Scientific #31331028) supplemented with 10% Fetal Bovine Serum (FBS, Dutscher #500101L), streptomycin (100 μg/ml), and penicillin (100 U/ml). The human mesothelial cell line MeT‐5A was purchased from ATCC (#CRL‐9444) and cultured in Medium 199 containing 1.5 g/l sodium bicarbonate (Sigma‐Aldrich #M4530), 10% FBS, 3.3 nM epidermal growth factor (EGF, Sigma‐Aldrich #E9644), 400 nM hydrocortisone (Sigma‐Aldrich #H0888‐1G), 870 nM zinc‐free bovine insulin (Sigma‐Aldrich #I9278), and 20 mM HEPES (Gibco #15630).

Cells were maintained at 37°C with 5% CO_2_ in the air atmosphere. They were routinely checked for mycoplasma (PlasmoTest™‐Mycoplasma Detection Kit, InvivoGen, #rep‐pt1) and underwent cell authentification by short tandem repeat analysis (powerplex16 HS kit, Promega #DC2101) processed at the Genomics Platform (Department of Translational Research, Institut Curie).

#### Immunofluorescence staining of centrosomes

##### Tissue sections

Frozen tissue sections of ovarian cancers and healthy tissues (20 μm of thickness) were fixed in cold methanol (−20°C) for 5 min and washed three times for 10 min in PBS 1X. Sections were permeabilized 10 min using PBS supplemented with 0.5% Triton X‐100, blocked 1 h in PBS + 0.3% Triton X‐100 + 3% of Bovine Serum Albumin (BSA). Tissues sections were incubated overnight at 4°C with primary antibodies diluted in PBS 1X + 0.3% Triton X‐100 + 3% BSA. Antibodies used: mouse anti‐pericentrin (1/250, Abcam #ab28144), rabbit anti‐CDK5RAP2 (1/500, Bethyl #BETIHC‐00063), rabbit anti‐CEP135 (generated in the lab—a MBP‐CEP135 fusion containing the first 493aa was used to immunize rabbits according to manufacturer's protocol‐Covalab, Lyon, France. After purification, described in (Vargas‐Hurtado *et al*, 2019), the antibody was used 1/500). For CEP192, we used the same strategy as described in (Vargas‐Hurtado *et al*, 2019) but guinea pig animals were immunized according to manufacturer's protocol—(Covalab, Lyon, France‐ antibodies 1/500). Sections were washed three times for 10 min in PBS 1X + 0.1% Triton X‐100 + 1% BSA and incubated for 6 h with secondary antibodies at 4°C: goat anti‐mouse IgG (H+L) highly cross‐adsorbed secondary antibody Alexa Fluor 568 (1/500, Invitrogen #A‐11031), goat anti‐rabbit IgG (H+L) highly cross‐adsorbed secondary antibody Alexa Fluor 488 (1/500, Invitrogen #A‐11008) and goat anti‐guinea pig IgG (H+L) highly cross‐adsorbed secondary antibody Alexa Fluor 647 (1/500, Invitrogen #A‐21450). After 3 × 10 min of washing in PBS 1X + 0.1% Triton X‐100 + 1% BSA, sections were mounted using Vectashield with DAPI mounting media (Vector Laboratories, #H‐1200).

##### Cell lines

Cells were fixed in cold methanol (−20°C) for 5 min, washed and permeabilized three times for 5 min using PBS‐T (PBS 1X + 0.1% Triton X‐100 + 0.02% Sodium Azide). Next, cells were blocked for 30 min at RT with PBS‐T supplemented with 0.5% BSA. Cells were incubated for 1 h at RT with primary antibodies diluted in PBT + 0.5% BSA. We used the same antibodies as described above. Cells were washed three times for 5 min and incubated for 30 min with secondary antibodies diluted in PBT + 0.5% BSA: goat anti‐mouse IgG (H + L) highly cross‐adsorbed secondary antibody Alexa Fluor 568 (1/500, Invitrogen #A‐11031), goat anti‐Rabbit IgG (H + L) highly cross‐adsorbed secondary antibody Alexa Fluor 488 (1/500, Invitrogen #A‐11008). Cells were washed 3 × 5 min and incubated for 10 min with DAPI (1/2,000, Invitrogen #D1306) diluted in PBT + 0.5% BSA. Finally, cells were washed the last three times in PBT + 0.5% BSA and once with PBS 1X, then mounted with a home‐made mounting medium.

#### Stable cell lines with PLK4 inducible overexpression

##### Generation of inducible cell lines

To generate PLK4‐inducible stable cell lines from OVCAR8 and SKOV3 cells, we used a doxycycline‐inducible PLK4 lentiviral expression system (Holland *et al*, 2012). Viruses were produced in HEK293T cells, co‐transfected with two other vector plasmids using lipofectamine 2000: a vesicular stomatitis virus envelope expression plasmid (Vsvg) and a second‐generation packaging plasmid (pPax2). Viral particles were then used to infect OVCAR8 and SKOV3 cell lines for 24 h. Infected cells were selected using bleomycin 50 μg/ml (Santa cruz Biotechnology #sc200134A) for 15 days. Newly generated stable cell lines iOVCAR8 and iSKOV3 were then expanded in DMEM/F12 media supplemented with 10% tetracycline‐free fetal bovine serum (FBS, Dutscher #S181T), streptomycin (100 μg/ml,), and penicillin (100 U/ml). To induce PLK4 overexpression, cells were treated with doxycycline (1 μg/ml) for 96 h.

##### Cell growth

10^5^ cells were plated per well in a 6‐well plate and treated after adhesion with centrinone, doxycycline, or corresponding controls. Living cells were trypsinized and counted at 24, 48, 72, and 96 h postseeding by Vi‐Cell analyzer (Beckman Coulter), using a trypan blue exclusion assay.

##### Cell death

10^5^ cells were plated per well in a 6‐well plate and treated with centrinone, doxycycline, or corresponding controls over 96 h. Next, cells were washed twice in cold PBS 1X and 100 μl of cells suspension were stained for 15 min with 5 μl of Annexin V APC and 10 μl of propidium iodide 0.5 mg/ml (PI), all furnished in the same kit (Biolegend #640932). Apoptotic (annexin V‐positive) and necrotic (PI‐positive) cells were detected using a flow cytometer (BD LSR II cytometer). FlowJo software was used to analyze results.

#### Rac1 activation assay

##### Pull‐down assay

We performed Rac1–GTP pull‐down assay using the Rac1 activation kit (# BK035‐S, Cytoskeleton) according to the manufacturer's instructions. After centrinone or doxycycline treatment (and corresponding controls), adherent cells were scrapped and collected in lysis buffer. Then, protein extracts were incubated with PAK‐PDB affinity beads. All the experiments were done at 4°C. Next, beads were washed and resuspended in Laemmli buffer for western blotting analysis. *Western blotting*: Proteins were separated on 4–20% SDS electrophoresis gel and transferred onto PVDF membranes using Trans‐Blot Turbo Transfer System (#1704156, Biorad). Images were acquired using Chemidoc Imaging system (Biorad) and band intensities were quantified using Image Lab 6.0.1 software (Biorad).

##### Transient depletion of centrosomes

For centrosome depletion, we used the centrinone drug previously described in Wong *et al* (2015) and now commercially available (Clinisciences #HY‐18682). Briefly, 10^5^ iOVCAR8 or iSKOV3 cells were plated per well in 6‐well plates and allowed to adhere for at least 4 h before centrinone treatment at 200 nM. Dimethyl sulfoxide (DMSO, Sigma‐Aldrich #D8418) alone, at equivalent concentrations (v/v), was used as a negative control. Cells were incubated at 37°C for 96 h.

Note that the number of centrosomes was quantified (as described in the quantification section) following DMSO or centrinone treatment, to verify the efficiency of the drug before all functional assays.

#### Spheroid‐induced mesothelial clearance assay and live cell imaging

Ovarian cancer cells were cultured for 24 h on standard culture plates and 72 h on Poly‐2‐HydroxyEthlylMethacrylate‐coated culture dishes (poly‐HEMA, Sigma #3932). Poly‐HEMA prevents the cells from attaching to the culture dish, allowing them to remain in suspension and form spheroids. Briefly, dishes were coated with poly‐HEMA at 12 mg/ml in 95% ethanol and 0.8 mg/cm^2^ of density, next dried overnight at 37°C and sterilized before the experiment using ultrapure water supplemented with streptomycin (100 μg/ml) and penicillin (100 U/ml). Then, poly‐HEMA‐coated dishes were washed three times with ultrapure water and PBS before 10^5^ cells were plated.

To form mesothelial cells monolayer, 2 × 10^5^ Met‐5A cells were plated in Ibidi μ‐Slide 8 Well (Clinisciences #80826) coated with collagen type I (Sigma‐Aldrich #C3867‐1VL) and incubated for 48 h at 37°C. Before imaging, Met‐5A monolayer were labeled with 5 μM of CellTracker™ Orange CMRA Dye (ThermoFisher Scientific #C34551) and cancer cell spheroids were labeled with 10 μM of CellTracker™ Green CMFDA Dye (ThermoFisher Scientific #C7025) for 30 min. The tumor cell spheroids were added to the mesothelial monolayer and allowed to attach for 30 min before imaging. The exclusion of mesothelial cells induced by tumor spheroids was analyzed by live imaging.

In parallel, a pool of tumor cell spheroids was dissociated using trypsin, transferred onto slides by cytocentrifugation (cytospin™, Thermo Fisher Scientific), and labeled with centrosome markers (as described above) to validate treatment efficiency (centrinone vs. DMSO) in decreasing centrosome numbers.

#### Basement membrane isolation

For animal care, we followed the European and French National Regulation for the Protection of Vertebrate Animals used for Experimental and other Scientific Purposes (Directive 2010/63; French Decree 2013‐118). Animals were housed and bred in the SPF animal facility of the Institut Curie. Mesentery BM was isolated from 5 months old female C57Bl6/N mice and glued (3M Vetbond) on 24‐well plate inserts (BD Biosciences) from which the polycarbonate membrane was previously removed. Those basement membranes (BM) were decellularized for 40 min into 1 M ammonium‐hydroxide (Sigma‐Aldrich). BM are sterilized O/N at 4°C with 4 μg/ml of ciprofloxacin (Panpharma) and 1.25 mg/ml of metronidazole (B. Braun) diluted into PBS. Mesenteries were then stored for up to 48 h at 4°C into PBS with 2% Antibiotic‐Antimycotic solution (ThermoFisher).

#### Invasion assays (BM) and staining

10^5^ iSKOV3 cancer cells were cultured for 48 h on standard culture plates and for 48 h on Poly‐HEMA‐coated 6‐well plates, in presence of centrinone at 200 nM or DMSO alone at an equivalent concentration (v/v).

The BM was placed into a well filled with DMEM‐F12 supplemented with 10% FBS, 2% antibiotic‐antimycotic, 10 mM Hepes (Thermo Fisher). On the top side of the mesentery, aggregates from one well of a 6‐well plate were plated in the presence of DMEM‐F12 with 2% antibiotic‐antimycotic, 10 mM Hepes. Cells were cultured for 7 days at 37°C and 5% CO_2_ in the presence of centrinone at 200 nM or DMSO at equivalent concentrations (v/v) added on both sides of the BM with each medium change after 3 days.

#### Immunofluorescence

Basement membrane was washed in PBS for 5 min and fixed with 4% PFA at RT. Cells were washed in PBS three times and stained for 2 h using Alexa Fluor™ 488 Phalloidin (1 unit/ml, A12379 ThermoFisher) and DAPI (1 μg/ml, D1306 ThermoFisher). BM was washed three times in PBS then mounted on glass‐bottom dishes using Polymount medium (Polysciences) on both sides of the mesentery.

#### Immunofluorescence microscopy

##### For tissue sections

###### Confocal microscopy

LSM Nikon A1r was used to obtain optical sections along the Z axis (60×, Z‐distance of 0.5 μm, NIS Element software) of ten random fields from the entire tissue section. Centrosomes were identified through the co‐localization of two centrosomes markers.

###### Super resolution microscopy

Images were acquired on a spinning disk microscope (Gataca Systems, France), through a 100× 1.4NA Plan‐Apo objective with a sCMOS camera (Prime95B, Photometrics, USA), z distance of 0.2 μm. Multi‐dimensional acquisitions were performed using Metamorph 7.10.1 software (Molecular Devices, USA). Super resolution was achieved on the CSU‐W1 spinning disk equipped with a super‐resolution module (Live‐SR, Gataca systems). Images are presented as maximum intensity projections generated with ImageJ software.

##### For cell lines

###### Fluorescence microscopy

Images were acquired on an upright widefield microscope (DM6B, Leica Systems, Germany) equipped with a motorized XY and a 100× objective (HCX PL APO 100×/1,40‐0,70 Oil from Leica). For each condition, optical sections of images were acquired with a Z‐distance of 0.3 μm (Metamorph software) from at least 10 random fields. Images are presented as maximum intensity projections generated with ImageJ software.

###### Live imaging

Images were acquired every 30 min over 12 h using a spinning disk microscope (20× objective, 2.5 μm of z sections, Gataca Systems, France). Images were presented as maximum intensity projections generated with ImageJ software.

#### Invasion assays

Cells were imaged with an inverted laser scanning confocal LSM 880 NLO (Zeiss, Jena, Germany) coupled with Argon 488 laser (GFP) and diode 405 (DAPI) using 25×/0,8NA oil‐immersion objectives (Zeiss). BM were imaged using second‐harmonic generation microscopy. Optical sections of images were acquired with a Z‐distance of 2.5 μm and treated using Imaris (Bitplane).

### Quantification and statistical analysis

#### Tumors

For each sample, 10 randomly chosen fields were considered. Using ImageJ software, we visually counted the number of nuclei and the number of centrosomes in a blind manner without taking into account tumor identity.

#### Quantification of the frequency of centrosome amplification events and the frequency of nuclei without centrosomes

Any group of more than two centrosomes including isolated centrosomes, clusters, and super‐clusters was considered as one centrosome amplification event. In regions of dense centrosomes and dense nuclei, where it was not possible to ascertain one centrosome or a group of centrosomes to a given nuclei and so to consider if it corresponds to centrosome amplification, were discarded for quantification. Considering nuclei without centrosomes, we only took into account, areas of the tissue containing nuclei that were separated enough from other nuclei to be able to distinguish nuclei without centrosomes. Regions where it was not possible to distinguish if one or more centrosomes belong to a particular nucleus were discarded for quantifications.

#### Quantification of centrosome number in cell lines

To quantify the percentage of cells with zero, one, two, or more than two centrosomes we used max z projections for each condition analyzed. At least 100 cells per condition were taken into account.

#### Mesothelial clearance quantification

The area—visible as a black region—induced by cancer cell spheroids invading into fluorescent mesothelial monolayer was analyzed every 30 min using ImageJ software. The area of the aperture size measured as readout of clearance was normalized by the initial spheroid size.

#### Invasion assay quantification

Analysis was performed using Imaris (BitPlane). The total number of nuclei was counted using the surface module. The BM was settled as the reference frame. Invaded nuclei were automatically counted as the object detected below the reference frame. Invasion frequency was calculated as the number of invading nuclei per the total number of nuclei per field obtained with the ×25 objective.

#### Statistical analysis

For mesothelial clearance, repeated‐measure analysis of covariance (ANCOVA) was used, since longitudinal measurement of clearance were balanced with evenly space–time points for all the conditions. The clearance (mean) according to the log of time (h) was plotted, then differences in slopes and intercepts among regression lines were evaluated. Differences were considered statistically significant at values of *P* ≤ 0.05.

## Author contributions

**Renata Basto:** Conceptualization; funding acquisition; validation; investigation; methodology; writing – original draft; project administration; writing – review and editing. **Jean‐Philippe Morretton:** Investigation; writing – original draft; validation. **Anthony Simon:** Conceptualization; resources; validation; investigation; visualization; methodology; writing – original draft; writing – review and editing. **Aurelie Herbette:** Investigation. **Jorge Barbazan:** Conceptualization; methodology. **Carlos Pérez‐González:** Conceptualization; methodology. **Camille Cosson:** Investigation. **Bassirou Mboup:** Methodology. **Aurelien Latouche:** Methodology. **Tatiana Popova:** Methodology. **Yann Kieffer:** Methodology. **Anne‐Sophie Macé:** Methodology. **Pierre Gestraud:** Methodology. **Guillaume Bataillon:** Resources; validation. **Véronique Becette:** Resources; validation. **Didier Meseure:** Resources; validation. **Andre Nicolas:** Resources; validation. **Odette Mariani:** Resources. **Anne Vincent‐Salomon:** Resources; validation. **Marc‐Henri Stern:** Resources; methodology. **Fatima Mechta‐Grigoriou:** Conceptualization; resources; validation; methodology. **Sergio Roman Roman:** Resources; funding acquisition. **Danijela Matic Vignjevic:** Conceptualization; validation; methodology. **Roman Rouzier:** Resources; supervision; funding acquisition; validation. **Xavier Sastre‐Garau:** Conceptualization; resources; funding acquisition; validation; investigation; methodology. **Oumou Goundiam:** Conceptualization; resources; supervision; funding acquisition; validation; investigation; methodology; writing – original draft; project administration; writing – review and editing.

In addition to the CRediT author contributions listed above, the contributions in detail are:

The project was initially designed and conceptualized by OG, XS‐G, and RB with significant input from SRR. J‐PM, and AS performed most experiments, including immunostaining, and centrosome number quantifications of all the tissues and cell lines, the clearance and invasion assays. CC was involved in setting up the protocols for centrosome analysis in cells and tissues. JB and CP‐G contributed to mesothelial clearance and invasion assays with significant input from DMV about cell invasion processes. A.H. helped establish the stable cell lines used for functional assays; A‐SM helped with the development of macros for clearance analysis. BM, AL, TP, M‐HS, PG, DM, AN, and YK, contributed with expertise and/or provided advice in statistical analysis. GB and VB did the pathological review of all the specimens; OG performed RAC1‐activation assays. OM managed tumor samples availability; XS‐G and AV‐S provided human samples from the pathology department of Institut Curie; RR supplied surgical pieces to enlarge the cohort and provided expertise in ovarian cancers. FM‐G provided the OVCAR8 cell line, and expertise in EOCs. SRR provided advice in the methodology used. The work was supervised by OG and RB. AS and RB prepared the figures and wrote the manuscript.

## Disclosure and competing interests statement

TP and M‐HS are co‐inventors of the LST method (US20170260588, US20150140122 and exclusive License to Myriad Genetics). The other authors declare no competing interests.

## For more information

i https://ocrahope.org/
ii https://ovarian.org.uk/
iii https://www.cancer.org/cancer/ovarian‐cancer.html
iv https://www.foundationforwomenscancer.org/gynecologic‐cancers/cancer‐types/ovarian/

## Supporting information



Expanded View Figures PDF

Table EV1

Source Data for Expanded View

## Data Availability

This study includes no data deposited in external repositories.
