# Supplementary material for: A catalog of numerical centrosome defects in epithelial ovarian cancers
Source: EMBO Mol Med. 2022 Sep 7;14(9):e15670. doi: 10.15252/emmm.202215670 (PMC9449595; doi:10.15252/emmm.202215670)
Supplement: Supplementary file 1 — Expanded View Figures PDF [file EMMM-14-e15670-s004.pdf]

Expanded View Figures

Figure EV1. Characterization of ovarian cancer stable cell lines after gain or loss of centrosomes.

A Scheme of the different experimental conditions and nomenclature used.

B Representative images of iOVCAR8 (top) and iSKOV3 (bottom) after the indicated treatments labeled with antibodies against PCNT and CDK5RAP2 (red and green respectively, DNA in blue). The white dashed squares represent the regions shown in higher magnification on the bottom.

C Graph bars representing the percentage of cells according to centrosome number after the different treatments of iOVCAR8 (left) and iSKOV3 (right), at least 80 cells analyzed by condition.

D Graphs representing the proliferation of iOVCAR8 and iSKOV3 cells after each treatment for 4 days. From three replicates in three independent experiments, statistical significances assessed with two-way ANOVA, $n = 6$ independent experiments. Mean ± SD.

E Graph bars representing the viability of iOVCAR8 and iSKOV3 cells after the designated treatment according to indicated timing in days. From three replicates in three independent experiments, statistical significance was assessed with one-way ANOVA, $n = 6$ independent experiments. Mean ± SD.

F On the left, graph bars represent the percentage of apoptotic cells iOVCAR8 and iSKOV3 after each indicated treatment. Statistical significances were assessed with the Wilcoxon test, $n = 6$ independent experiments (H). Mean ± SD. On the right, representative FACS plots showing Annexin V^+ (*x*-axis) and PI^+ (*y*-axis) cells, for iOVCAR8 (top) and iSKOV3 (bottom) cells after the indicated treatments. For all experiments, $n = 3$ independent experiments.

G, H Western blot and graphs bars representing the quantifications of active RAC-1 in iOVCAR8 and iSKOV3 cell lines after the indicated treatments. Statistical significances were assessed with one-way ANOVA, $n = 6$ independent experiments. Mean ± SD.

I, J Dot plot graphs showing the percentage of cells in each category according to centrosome number corresponding to the experiments of mesothelial cell clearance (I) and BM invasion assays (J). At least 80 cells were analyzed per condition.

Source data are available online for this figure.

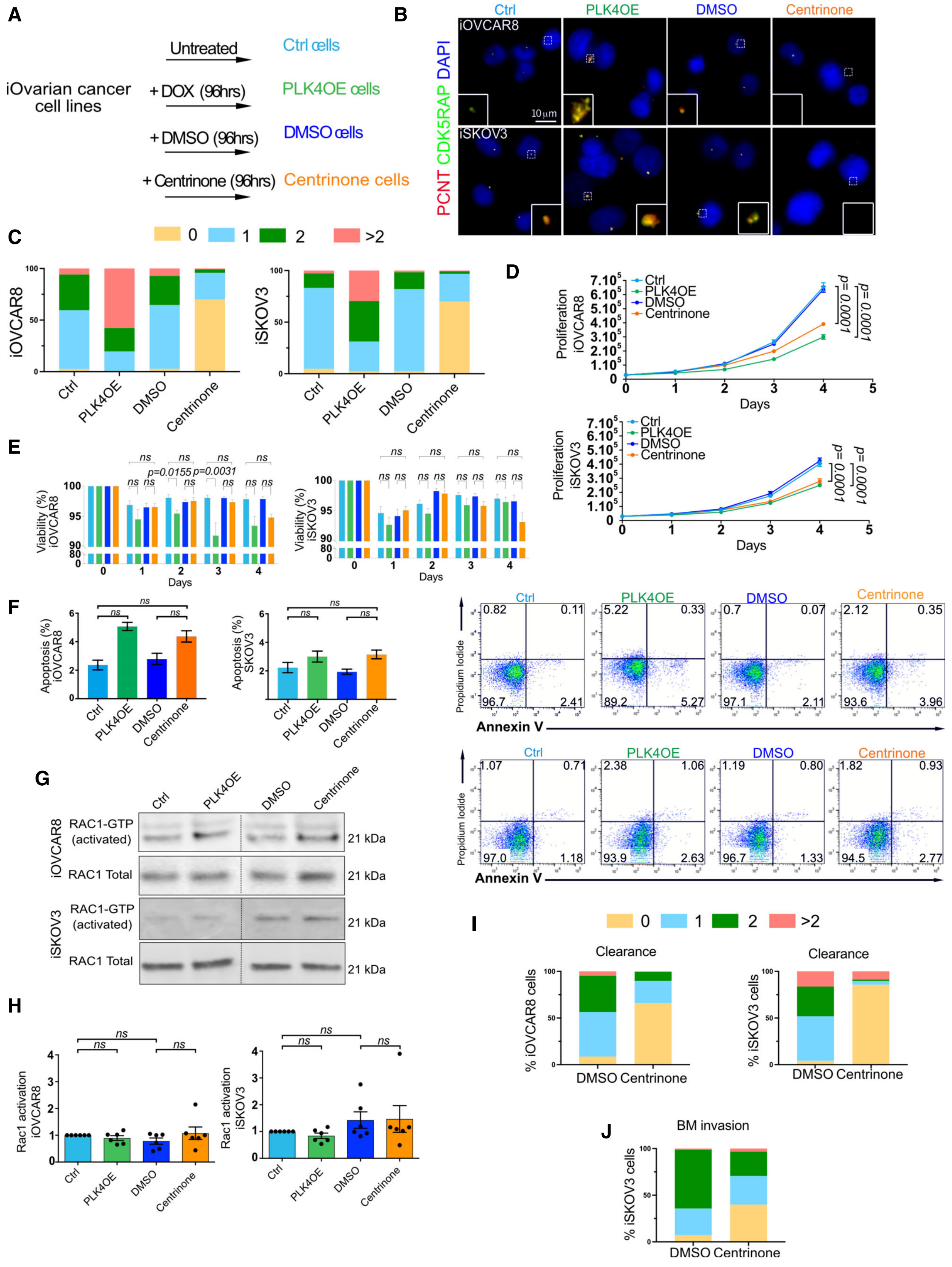

Figure EV1.

© 2022 The Authors
