# Supplementary material for: A catalog of numerical centrosome defects in epithelial ovarian cancers
Source: EMBO Mol Med. 2022 Sep 7;14(9):e15670. doi: 10.15252/emmm.202215670 (PMC9449595; doi:10.15252/emmm.202215670)
Supplement: Supplementary file 2 — Table EV1 [file EMMM-14-e15670-s001.pdf]

Table EV1

Samples from Institut Curie	
**Ovarian healthy samples**	**19 samples**
**Ovarian tumor samples (1990-2012):**	**100 samples**
Primary tumors (samples after surgery and before chemotherapy)	100%
**Age at diagnosis (years):**	
Median	60
Range	31-87
**Grades:**	
Low	3 (3%)
High	97 (97%)
**FIGO stages:**	
I	10(10%)
II	8 (8%)
III	59(59%)
IV	12 (12%)
NA	11 (11%)
**Histological types:**	
Serous	90 (90%)
Endometrioid	3 (3%)
Mucinous	4 (4%)
Clear cells	3 (3%)
**Nb of tumors with genomic data (Affymetrix Cytoscan Array)**	81 (81%)

Clinical data of the patients included in this study and corresponding tumor characterization

Clinical data, including FIGO staging, histology type, grade and age at diagnosis of the patients included in this study were prospectively registered.
