# Supplementary material for: A catalog of numerical centrosome defects in epithelial ovarian cancers
Source: EMBO Mol Med. 2022 Sep 7;14(9):e15670. doi: 10.15252/emmm.202215670 (PMC9449595; doi:10.15252/emmm.202215670)
Supplement: Supplementary file 4 [file EMMM-14-e15670-s002.pdf]

EMBO Molecular Medicine

A catalog of numerical centrosome defects in epithelial ovarian cancers

Renata Basto, JP Morretton, Anthony Simon, Aurelie Herbette, Jorge Barbazan, Carlos Perez-Gonzalez, Camille Cosson, Bassirou Mboup, Aurelien Latouche, Tatiana Popova, Yann Kieffer, Anne-Sophie Macé, Pierre Gestraud, Guillaume Bataillon, Véronique Becette, Didier Meseure, Andre Nicolas, Odette Mariani, Anne Vincent-Salomon, Marc-Henri Stern, Fatima Mechta-Grigoriou, Sergio Roman-Roman, Danijela Vignjevic, Roman Rouzier, Xavier Sastre-Garau, and Oumou Goundiam
DOI: 10.15252/emmm.202215670

Corresponding authors: Renata Basto (renata.basto@curie.fr) , Oumou Goundiam (oumou.goundiam@curie.fr)

Review Timeline:

Submission Date:	3rd Jan 22
Editorial Decision:	28th Jan 22
Revision Received:	26th Apr 22
Editorial Decision:	30th May 22
Revision Received:	16th Jun 22
Editorial Decision:	24th Jun 22
Revision Received:	13th Jul 22
Editorial Decision:	15th Jul 22
Revision Received:	21st Jul 22
Accepted:	21st Jul 22

Editor: Zeljko Durdevic

Transaction Report:

(Note: With the exception of the correction of typographical or spelling errors that could be a source of ambiguity, letters and reports are not edited. Depending on transfer agreements, referee reports obtained elsewhere may or may not be included in this compilation. Referee reports are anonymous unless the Referee chooses to sign their reports.)

28th Jan 2022

Dear Dr. Basto,

Thank you for the submission of your manuscript to EMBO Molecular Medicine. We have now received feedback from the two reviewers who agreed to evaluate your manuscript. As you will see from the reports below, the referees acknowledge the interest of the study but also raise important concerns that should be addressed in a major revision.

Further consideration of a revision that addresses reviewers' concerns in full will entail a second round of review. EMBO Molecular Medicine encourages a single round of revision only and therefore, acceptance or rejection of the manuscript will depend on the completeness of your responses included in the next, final version of the manuscript. For this reason, and to save you from any frustrations in the end, I would strongly advise against returning an incomplete revision.

We would welcome the submission of a revised version within three months for further consideration. Please let us know if you require longer to complete the revision.

Please use this link to login to the manuscript system and submit your revision: https://embomolmed.msubmit.net/cgi-bin/main.plex

I look forward to receiving your revised manuscript.

Yours sincerely,

Zeljko Durdevic

Zeljko Durdevic
Editor
EMBO Molecular Medicine

***** Reviewer's comments *****

Referee #1 (Remarks for Author):

Centrosome abnormalities, in particular amplification of centrosome numbers, have been associated with multiple human tumours. Several studies using human tissue clearly showed that specific types of breast cancer have indeed increased centrosome numbers, which are rarely observed in healthy tissue. These abnormalities have been associated with CIN and poor prognosis but compelling data is snot abundant. In addition, the frequency of such anomalies within a tumour is also not well established and it has been observed cells with extra centrosomes may be exist in lower numbers. Interestingly, prostate tumours contain cells without centrosomes that are associated with tumour progression. Thus, it is clear that the impact of centrosome numerical abnormalities to tumourigenesis will depend on the context. This is supported by work using mouse tumour models (including seminal work from the lead author of this manuscript) where centrosome amplification may have opposite effects depending on tissue type.

In this manuscript, Morretton et al. uses ovarian cancer as a model to characterise centrosome abnormalities in humans. They define a 'CNI' and found that, unlike healthy tissues, ~60% of the analysed ovarian tumours contain increased centrosome numbers. The CNI however seems in general low, suggesting that centrosome amplification is not widespread but rather observed in some cells. Furthermore, these tumours also contain cells, areas where centrosomes are lost, which can further decrease the CNI. By separating low CNI and high CNI tumours, the authors found that low CNI is associated with poor prognosis, especially in HRP tumours, suggesting this index could be used to stratify patients. The poor prognosis of low CNI tumours could be associated with mesenchymal signatures, previously associated with worse outcome.

This work is a true tour de force. Defining centrosome numbers in tumours is a rather difficult task and comprehensive studies are lacking, which this study provides for ovarian cancer. Overall, the experiments are well conducted and presented. In my opinion, this work would generate interest to the wider scientific community. My comments, outlined below, are related with the definition of CNI and as a consequence the interpretation of some of the findings. I think these points could be better explained/ taken into account by the authors as it does not have to be always black or white.

Major comments:

1. Definition of CNI. Centrosome-nucleus-index (CNI) is defined by the authors as a measure of centrosome amplification using PCM markers. Quantifying numbers of centrosomes per cell within tumours is very difficult and I appreciate that better systems may not exists. However, this come with limitations that need to be outlined in the manuscript more clearly: effective centriole numbers are not quantified in tumours, this could impact analyses; and populations analyses could undermine the real % of cells with centrosome abnormalities, as the authors find out in fig2C, D, where the presence of pockets of cells without centrosomes will overall decrease CNI, even in the presence of cells with amplified centrosomes.

2. Definition of low CNI. Because healthy tissues have low CNI, low CNI is the normal condition. This for me complicates interpretation because it is easy to assume from the title of this manuscript that low CNI means low centrosome numbers. But that is not always the case. A likely better way to divide these tumours would have been: low CNI < 1 (abnormal), normal CNI 1-1.3 (normal) and higher CNI> 1.3 (abnormal) (For me it is not clear why CNI 1-1.5 is considered low if healthy tissues never go above 1-1.2?). However, low CNI is not always equivalent and thus not always mean normal centrosome numbers. Basically, in tumours with low CNI there will be tumours without centrosome abnormalities, just like healthy tissues, tumours with very little levels of extra centrosomes and tumours with cells with high levels of extra centrosomes and cells without centrosomes (as in fig 2C-D). Thus, this category is rather heterogeneous. I understand that it may be difficult to divide the tumours in smaller categories as this will decrease sample number, but, for the purpose of assessing the impact of CA, should the tumours identified in fig 2C with low CNI but with similar number of areas of CA to high CNI be included in the high CNI? Or should the tumours with areas that lack centrosomes be included in a separate category: low centrosome numbers and analysed

separately?

3. Related with the above comments, does the presence of superclusters in the low CNI (as seen in suppl fig 1B) impact outcome of tumours? Or it does not matter?

Referee #2 (Remarks for Author):

Centrosomes are microtubule-organizing centers that function in bipolar spindle formation during mitosis and primary cilia formation during interphase. Centrosome number is tightly controlled to ensure the fidelity of chromosome segregation and centrosome gains or losses have been linked to aneuploidy, cancer, and microcephaly. While several pieces of work have demonstrated that centrosome amplification causes tumorigenesis in mice and flies, most of the work on centrosome amplification has been performed in cultured cell lines. Thus, there is a strong need for careful analysis of clinical tumors.

In this paper, Morretton et al confront this challenge by evaluating tissue samples from individuals with ovarian cancer. They use the well-established centrosome markers (PCNT and CDK5RAP2) to label centrosomes and find that the tumor samples are very heterogenous showing regions of centrosome loss and other cells with centrosome amplification. They develop a metric to classify centrosome number in tumors called CNI and then go on to try to correlate CNI with molecular and clinical characteristics of the tumors. They find no correlation between CNI and proliferation or chromosomal structural abnormalities. Moreover, invasion and migration of cultured ovarian cancer cell lines are unaffected with changes in centrosome number. However, they do find worse patient survival in those with low CNI tumors, perhaps due to enrichment of mesenchymal tumor subtypes.

In general, this work is important to the field; however, several important issues outlined below need to be addressed.

1. Given the technical difficulty of these experiments, the staining and microscopy is impressive. However, the characterization of centrosomes in tumor samples is very descriptive and lacks proper quantitation. It would be helpful to understand what percentage of cells in a tumor have centrosome loss compared to centrosome amplification. This is especially important given the high heterogeneity among different sections from the same tumor.
2. The CNI metric is troubling for several reasons. First, because cells in tumor samples are disorganized compared to control tissues, how confident can the authors be that each tissue section encompasses an entire cell without a cell membrane marker? In other words, how likely is it that centrosome counts are over or underestimated due to centrosomes being outside the z-stack while the nucleus is within the stack (or vice versa). Perhaps this could be solved by analyzing centrosome numbers in a couple samples that include a cell membrane marker to demonstrate that similar centrosome numbers are observed as with CNI. Second, it is unclear how the authors determine "normal CNI". Presumably a normal CNI would be 1 centrosome per cell as found in the healthy tissue samples. However, the authors have set a value of 1.45 as "normal CNI". This seems arbitrary and leads to defining a large set of tumor samples as having low CNI when in fact the CNI of these tumor samples is no different than the healthy tissues. In my interpretation of Fig. 3B, most tumor samples have high CNI; however, the authors come to the opposite conclusion and then spend much of the later figures trying to correlate CNI with molecular and clinical parameters. I understand that accounting for centrosome clusters implies more cells in the low CNI category lack centrosomes, but without rigorous quantitation as suggested above, this is too indirect. Overall, the CNI metric seems too broad resulting in loss of important details about centrosome number per cell.
3. Similarly, the title is misleading along with some conclusions of this paper. Low centrosome number implies (in my interpretation at least) less centrosomes than control/healthy cells. But the authors have not directly demonstrated decreased centrosome number in low CNI samples. In fact, a low CNI suggests similar centrosome number as control cells. Perhaps the authors could perform more rigorous quantitation as suggested above or focus on their findings that tumors with centrosome amplification have better clinical outcomes.
4. Is there any correlation with centrosome clusters/super clusters and nuclei size/ploidy?

Rebuttal letter- revision of EMM-2022-15670

Referee #1 (Remarks for Author):

Centrosome abnormalities, in particular amplification of centrosome numbers, have been associated with multiple human tumours. Several studies using human tissue clearly showed that specific types of breast cancer have indeed increased centrosome numbers, which are rarely observed in healthy tissue. These abnormalities have been associated with CIN and poor prognosis but compelling data is snot abundant. In addition, the frequency of such anomalies within a tumour is also not well established and it has been observed cells with extra centrosomes may be exist in lower numbers. Interestingly, prostate tumours contain cells without centrosomes that are associated with tumour progression. Thus, it is clear that the impact of centrosome numerical abnormalities to tumourigenesis will depend on the context. This is supported by work using mouse tumour models (including seminal work from the lead author of this manuscript) where centrosome amplification may have opposite effects depending on tissue type.

In this manuscript, Morretton et al. uses ovarian cancer as a model to characterise centrosome abnormalities in humans. They define a 'CNI' and found that, unlike healthy tissues, ~60% of the analysed ovarian tumours contain increased centrosome numbers. The CNI however seems in general low, suggesting that centrosome amplification is not widespread but rather observed in some cells. Furthermore, these tumours also contain cells, areas where centrosomes are lost, which can further decrease the CNI. By separating low CNI and high CNI tumours, the authors found that low CNI is associated with poor prognosis, especially in HRP tumours, suggesting this index could be used to stratify patients. The poor prognosis of low CNI tumours could be associated with mesenchymal signatures, previously associated with worse outcome.

This work is a true tour de force. Defining centrosome numbers in tumours is a rather difficult task and comprehensive studies are lacking, which this study provides for ovarian cancer. Overall, the experiments are well conducted and presented. In my opinion, this work would generate interest to the wider scientific community. My comments, outlined below, are related with the definition of CNI and as a consequence the interpretation of some of the findings. I think these points could be better explained/taken into account by the authors as it does not have to be always black or white.

We thank this reviewer for his/her appreciation and acknowledgement that our work is relevant for the fields of centrosome and cancer biology. We also recognize that results and in particular results in the cell biology of cancer can be very heterogeneous and difficult to interpret. Certainly, we do not want to overstate our conclusion to paint a misleadingly black-and-white scenario. We hope that the reviewer will find this new version of the manuscript sufficiently nuanced.

Major comments:

1. Definition of CNI. Centrosome-nucleus-index (CNI) is defined by the authors as a measure of centrosome amplification using PCM markers. Quantifying numbers of centrosomes per cell within tumours is very difficult and I appreciate that better systems may not exists. However, this come with limitations that need to be outlined in the manuscript more clearly: effective centriole numbers are not quantified in tumours, this could impact analyses; and populations analyses could undermine the real % of cells with centrosome abnormalities, as the authors find out in fig2C, D, where the presence of pockets of cells without centrosomes will overall decrease CNI, even in the presence of cells with amplified centrosomes.

The reviewer is right, we defined centrosomes numbers using the CNI signature, which is an overview or global view of the centrosome number at the level of the population. But still by its definition, CNI = centrosome number/nuclei number, if there are many cells with more than two centrosomes, this will be observable in a higher CNI. We state this more clearly in the new version of the manuscript- We have included this in the abstract and in the results section. It is a very good suggestion to make reference to the evaluation of CNI representing a view of centrosome numbers at the level of the population.

Considering centriole markers, we understand this criticism very well. Initially, we focused on the PCM markers because we could buy the tools required to evaluate them and obtained large amounts, compared to the limited availability of homemade antibodies. In addition, the signal to noise in these 20μm sections was also very good with these two antibodies. To nevertheless take into account this point, we have used centriole markers and the co-localization of CEP192, CEP135 (using homemade antibodies) and PCNT. We had tested these antibodies before and used them in structural illumination microscopy in ovarian tissues as shown in Figs 1C and 2D. However, we could not have used them to characterize the 100 tumors as we did with the combination of CDK5RAP2 and PCNT. The main reason related with the fact that both CEP135 and CEP192 antibodies, for a reason that we do not know, give high levels of background in many tumor sections. This is a problem when quantifying centrosomes. To validate the quantification of centrosome numbers with PCM markers, we labeled 23 different tumor sections and we show that this leads to CNIs very close to the ones described before. We managed to obtain only 23 tumors

from the Institut Curie biobank corresponding to the tumors characterized previously. We think this is partially explained by the fact that PCNT mimics very closely the behavior of centrioles. We thus conclude and that our initial estimation was not an underestimation of centrosome number. We have included this data in Figure EV2F-G.

2. Definition of low CNI. Because healthy tissues have low CNI, low CNI is the normal condition. This for me complicates interpretation because it is easy to assume from the title of this manuscript that low CNI means low centrosome numbers. But that is not always the case. A likely better way to divide these tumours would have been: low CNI < 1 (abnormal), normal CNI 1-1.3 (normal) and higher CNI> 1.3 (abnormal) (For me it is not clear why CNI 1-1.5 is considered low if healthy tissues never go above 1-1.2?). However, low CNI is not always equivalent and thus not always mean normal centrosome numbers. Basically, in tumours with low CNI there will be tumours without centrosome abnormalities, just like healthy tissues, tumours with very little levels of extra centrosomes and tumours with cells with high levels of extra centrosomes and cells without centrosomes (as in fig 2C-D). Thus, this category is rather heterogeneous. I understand that it may be difficult to divide the tumours in smaller categories as this will decrease sample number, but, for the purpose of assessing the impact of CA, should the tumours identified in fig 2C with low CNI but with similar number of areas of CA to high CNI be included in the high CNI? Or should the tumours with areas that lack centrosomes be included in a separate category: low centrosome numbers and analysed separately?

We understand the different questions related with the CNI. The reviewer questions whether this was the right approach to classify these tumors. It is important to consider the following. Healthy tissues have a CNI of 1.02, which is expected from tissues with low proliferation potential. However, even without any centrosome defect, a proliferative cell should have a CNI of 2 from the largest part of its cell cycle- S-phase, G2 and Mitosis. It was thus surprising that the CNI in tumors (which are highly proliferative), is so low. So the CNI in tumors is very unexpected for a highly proliferative tissue.
The reviewer suggestion is quite interesting, but we feel that considering tumors with CNI of 1-1.2 as normal tissue is not correct. Several of these tumors have nuclei without centrosomes and a few nuclei with too many. So we would feel that this is passing on a wrong message. Also, there is no tumor without centrosome abnormality, even tumors with CNI around 1. To explain this point better, and to obtain a more detailed view of centrosome abnormalities in the tumors, we have re-analyzed all tumor sections from all the 100 tumors. We quantified the number of centrosome amplification events taking into consideration every condition with more than two centrosomes and so including isolated, clusters and super-clusters. As seen in the new graph of Figure 3D, the frequency of centrosome amplification was extremely variable and only reached a maximum of ~3% in a low number of tumors. Moreover, we also determined the frequency of nuclei that contained zero

centrosome. This was much higher than centrosome amplification (Figure 3E). With these graphs, we show the highly heterogeneous status of centrosome numbers. Further, we also show that absence of centrosome is a more frequent feature than centrosome amplification.

3. Related with the above comments, does the presence of superclusters in the low CNI (as seen in suppl fig 1B) impact outcome of tumours? Or it does not matter?

We have investigated this point by analyzing the impact of centrosome amplification including isolated, clusters and super-clusters on patient's overall survival. As seen in the graphs below, we found no association between outcome and the presence of centrosome amplification considering the four groups indicated thereafter (Log rank test, pvalue = 0.1315). For each group of Low or High CNI tumors, the curves with centrosome amplification show the same trend as those without centrosome amplification, without any striking significant dichotomization (please see the plot below, centrosome amplification= CA).

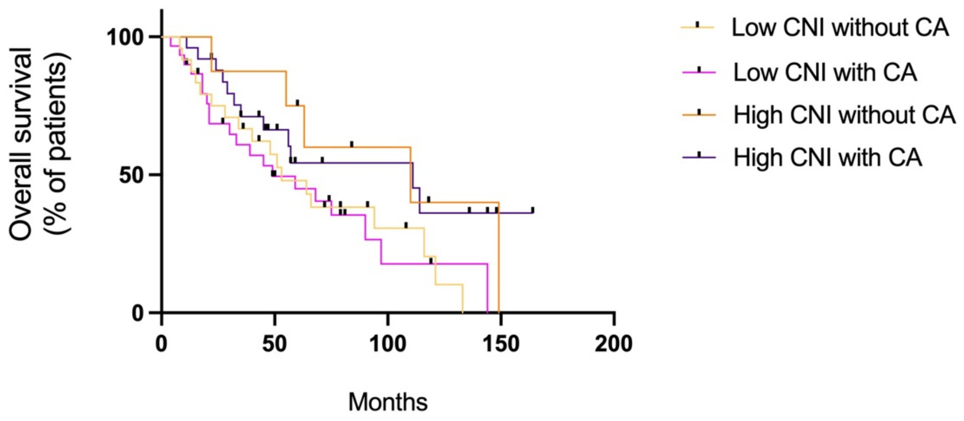

Referee #2 (Remarks for Author):

Centrosomes are microtubule-organizing centers that function in bipolar spindle formation during mitosis and primary cilia formation during interphase. Centrosome number is tightly controlled to ensure the fidelity of chromosome segregation and centrosome gains or losses have been linked to aneuploidy, cancer, and microcephaly. While several pieces of work have demonstrated that centrosome amplification causes tumorigenesis in mice and flies, most of the work on centrosome amplification has been performed in cultured cell lines. Thus, there is a strong need for careful analysis of clinical tumors.

In this paper, Morretton et al confront this challenge by evaluating tissue samples from individuals with ovarian cancer. They use the well-established centrosome markers (PCNT and CDK5RAP2) to label centrosomes and find that the tumor samples are very heterogenous showing regions of centrosome loss and other cells

with centrosome amplification. They develop a metric to classify centrosome number in tumors called CNI and then go on to try to correlate CNI with molecular and clinical characteristics of the tumors. They find no correlation between CNI and proliferation or chromosomal structural abnormalities. Moreover, invasion and migration of cultured ovarian cancer cell lines are unaffected with changes in centrosome number. However, they do find worse patient survival in those with low CNI tumors, perhaps due to enrichment of mesenchymal tumor subtypes.

In general, this work is important to the field; however, several important issues outlined below need to be addressed.

We thank the reviewer for his recognition of this work's importance, for the centrosome and cancer biology fields.

1. Given the technical difficulty of these experiments, the staining and microscopy is impressive. However, the characterization of centrosomes in tumor samples is very descriptive and lacks proper quantitation. It would be helpful to understand what percentage of cells in a tumor have centrosome loss compared to centrosome amplification. This is especially important given the high heterogeneity among different sections from the same tumor.

We understand very well this request. However, we can not ascertain with confidence the exact corresponding centrosomes to each nucleus in a section. We have to strengthen the point that these tissues are highly heterogeneous and disorganized, typical of ovarian cancers. This is why we took the strategy of quantifying the CNI. To nevertheless address this point, we have now quantified for each section for all the tumors the number of events with centrosome amplification, including isolated centrosomes, cluster and super clusters. We also analyzed the frequency of nuclei without centrosomes. These data are now included in the new figure 3D-E of the manuscript and described in the results. Indeed, we found that loss of centrosomes is more frequent than centrosome amplification in all the 100 tumors analyzed. Importantly, none of these defects - neither centrosome amplification nor loss - has ever been seen in healthy tissues.

2. The CNI metric is troubling for several reasons. First, because cells in tumor samples are disorganized compared to control tissues, how confident can the authors be that each tissue section encompasses an entire cell without a cell membrane marker? In other words, how likely is it that centrosome counts are over or underestimated due to centrosomes being outside the z-stack while the nucleus is within the stack (or vice versa). Perhaps this could be solved by analyzing centrosome numbers in a couple samples that include a cell membrane marker to demonstrate that similar centrosome numbers are observed as with CNI.
Second, it is unclear how the authors determine "normal CNI". Presumably a normal CNI would be 1 centrosome per cell as found in the healthy tissue samples.

However, the authors have set a value of 1.45 as "normal CNI". This seems arbitrary and leads to defining a large set of tumor samples as having low CNI when in fact the CNI of these tumor samples is no different than the healthy tissues. In my interpretation of Fig. 3B, most tumor samples have high CNI; however, the authors come to the opposite conclusion and then spend much of the later figures trying to correlate CNI with molecular and clinical parameters. I understand that accounting for centrosome clusters implies more cells in the low CNI category lack centrosomes, but without rigorous quantitation as suggested above, this is too indirect. Overall, the CNI metric seems too broad resulting in loss of important details about centrosome number per cell.

We understand this reviewer's concern considering the CNI quantifications. His/her suggestion of using a membrane marker has been considered. We tested several markers, and the best marker that worked on tissue preparations with protocols that allowed to label centrosomes, membranes and nuclei were antibodies against EpCAM. Nevertheless, the abnormalities in terms of membrane invagination and roughness were so frequent that this only further increased the difficulty of correlating centrosomes to a given nuclei. Indeed, while in certain "more organized tissue sections", we could clearly identify membrane boundaries, in the large majority of cases this was not possible. It is important to note that for the majority of the tissue section labelled with the EpCAM antibody, the signal was weak, or did not label certain areas of the tumor. For these reasons, we could not use it as a robust membrane marker of our tumor cohort. We spent time at the beginning of this project trying to obtain this type of labeling but had to give up. We have added this information to the text and included a picture in Figure EV2B to illustrate this point.

Considering the point related with losing centrosomes or nuclei. We have estimated the size of ovarian cells on average as 8.33± 2.1μm. We have done this, because,initially we worked with tissue sections of 3 and 5μm and we then noticed that we might been missing a proportion of cellular structures. That is why we then decided to obtain larger sections (20μm). As we go through Z sections from confocal microscopy, we are confident that we start from one extreme end and finish at the other. And we have always analyzed the sections individually but the quantifications were made on full Z-projections. We realize that we have not explained any of these details. We have now included this information in the main text and methods section (Figure EV2A).

To address now the point related with low CNI being similar to healthy tissues. This is not the case, as in healthy tissues all nuclei have one centrosome. We have never observed zero centrosome or extra centrosomes in healthy tissues, which are thus very different from what we have observed in tumors.

The choice of 1.45 was not arbitrary. It took into consideration the numbers of centrosome and nuclei as a whole population for every tumor. We have explained more in detail the choice of 1.45 and its validation in the text, page 11 and new Figure 3C. Still, we have addressed the point by categorizing the number of nuclei with extra centrosomes and without centrosomes. This is now presented in Figure 3D-E. Also the reviewer mentions that CNI=1 is what is considered normal. This is certainly the case in healthy tissue but it is not expected that a tumor has a CNI=1, since a large proportion of cells will be proliferating and so, when in S-G2-M, should have two centrosomes.

3. Similarly, the title is misleading along with some conclusions of this paper. Low centrosome number implies (in my interpretation at least) less centrosomes than control/healthy cells. But the authors have not directly demonstrated decreased centrosome number in low CNI samples. In fact, a low CNI suggests similar centrosome number as control cells. Perhaps the authors could perform more rigorous quantitation as suggested above or focus on their findings that tumors with centrosome amplification have better clinical outcomes.

We think that the definition of low CNI is to be taken into account at the level of the tumor population. The CNI of 1 in healthy tumors is explained simply by their low proliferative status. A cell that is proliferating should have 2 centrosomes, and so according to our definition of CNI, a CNI=2. And the surprising result is that this is not the case for the large majority of the samples. So this mean that cells proliferate (and they do, as we have quantified mitosis and Ki67 profiles) with less than two centrosomes/nuclei.
We have explained this aspect better and we have also called the reader’s attention to the important point that we are analyzing centrosomes at the level of the whole population.

Considering the title, we have changed for a broader title: A novel signature of ovarian cancers defined by centrosome number. We hope the reviewer prefers this title.

4. Is there any correlation with centrosome clusters/super clusters and nuclei size/ploidy?
In the previous version of the manuscript, we analyzed this point by ascertaining the tumors according to their CNI and ploidy (near diploid or tetraploid). There was no statistical difference. But we also agree with the reviewer that this is really an important point. So, in addition to the data mentioned above, we have analyzed the nuclear area of 756 nuclei from 21 tumors and assigned categories according to the centrosome number (zero, 1-2 or more than 2). We carefully chose the nuclei to be analyzed as we had to be sure that they were sufficiently isolated and well positioned to ascertain the position of centrosomes. As shown in the new Figure EV3H, there is

an enrichment for the largest nuclei for the >2 centrosome condition. But large nuclei can also be identified in the other categories. Once more, there is no statistical significance. So we think that there is a tendency to have larger nuclei associated with centrosome amplification, but larger nuclei can also be found associated with zero or 1 or 2 centrosomes.

30th May 2022

Dear Dr. Basto,

Thank you for the submission of your revised manuscript to EMBO Molecular Medicine. We have now heard back from the two referees who we asked to re-evaluate your manuscript. As you will see from the reports below, both referees acknowledge the improvements of the revised manuscript but also raise important and partially overlapping concerns that should be addressed in an additional and final round of major revision. Particular attention should be given to providing more detailed explanation of the CNI metric and its limitations.

Further consideration of a revision that addresses reviewer's concerns in full will entail an additional round of review. Acceptance or rejection of the manuscript will depend on the completeness of your responses included in the next, final version of the manuscript. For this reason, and to save you from any frustrations in the end, I would strongly advise against returning an incomplete revision.

We would welcome the submission of a revised version within three months for further consideration. Please let us know if you require longer to complete the revision.

Please use this link to login to the manuscript system and submit your revision: Link Not Available

I look forward to receiving your revised manuscript.

Yours sincerely,

Zeljko Durdevic

Zeljko Durdevic
Editor
EMBO Molecular Medicine

***** Reviewer's comments *****

Referee #1 (Remarks for Author):

The authors did a great addressing the comments raised. I agree with the new title and new descriptions added as it moves away from centrosome amplification which was a bit confusing.

The CNI as an index has its limitations and these must be outlined clearly. Most limitations in my opinion are not easily addressed and not sure more experiments would be valuable. The main limitation is the low correlation between centrosome numbers and CNI, which can now be seen in the new graphs in Figure 3D and 3E. The new data makes it clear that low and high CNI tumours do not differ much in terms of centrosome amplification, which is low in these tumours.

That said, it would be great is the authors could explain further the CNI based on the new data presented. How can some tumours have high CNI but almost no centrosome amplification and conversely, how tumours with the lowest CNI have only small percentage of cells with no centrosomes? It would be great if the authors could help understanding these discrepancies.

Referee #2 (Comments on Novelty/Model System for Author):

This work analyzes centrosome numbers in primary tumors. Something that has rarely been done.

Referee #2 (Remarks for Author):

The authors have clearly made an effort to incorporate reviewer comments into the revised manuscript. I consider the characterization of centrosome numbers in patient tumour samples a critical step for the field that fills a significant gap. My major problem with the original version of this paper was the use of the CNI metric. Unfortunately, the additional data presented in the revised version of this manuscript has only reinforced my initial concern. Fig 3 is especially problematic. Consider the tumour with the highest CNI: TT21. This tumour shows almost no centrosome amplification and has a 3% frequency of nuclei without centrosomes. Thus, I cannot understand how a tumour can have a high CNI without centrosome amplification? Conversely, the tumour with the lowest CNI is TT61. The tumour shows almost no centrosome amplification, and only 14% of nuclei do not have centrosomes. Given that the CNI of this tumour is ~0.6, shouldn't more than 14% of cells demonstrate centrosome loss to approach a CNI of about half? Mathematically this doesn't add up. While these two examples are of the tumours with the lowest and highest CNI, all tumours have a poor correlation of CNI with centrosome gain or loss.

The rest of the paper characterizes a subset of tumours based on CNI, and conclusions are drawn between centrosome number and clinical outcome. However, the authors have failed to demonstrate a correlation between CNI and centrosome numbers. Would better correlations occur if the authors analyzed tumours with the highest and lowest frequency of centrosome amplification/loss?

This is an important study analyzing centrosome numbers in vivo. I understand it is extremely challenging, and tumour samples are highly heterogeneous, but the current data analysis is insufficient to draw conclusions about centrosome numbers in ovarian tumour samples.

Response to reviewers- Revision 2- EMM-2022-15670-V2
Morretton and Simon, et al

Reviewers comments are in black and the author's response in green.

Referee #1 (Remarks for Author):

The authors did a great addressing the comments raised. I agree with the new title and new descriptions added as it moves away from centrosome amplification which was a bit confusing.

The CNI as an index has its limitations and these must be outlined clearly. Most limitations in my opinion are not easily addressed and not sure more experiments would be valuable. The main limitation is the low correlation between centrosome numbers and CNI, which can now be seen in the new graphs in Figure 3D and 3E. The new data makes it clear that low and high CNI tumours do not differ much in terms of centrosome amplification, which is low in these tumours.

That said, it would be great is the authors could explain further the CNI based on the new data presented. How can some tumours have high CNI but almost no centrosome amplification and conversely, how tumours with the lowest CNI have only small percentage of cells with no centrosomes? It would be great if the authors could help understanding these discrepancies.

We are extremely happy to find that this reviewer considers that we did a great job addressing the initial comments. Nevertheless, we are sorry to find out that this version still did not comply with the requirement of describing the CNI with its limitations. The reviewer also mentions the lack of correlation between the frequencies of centrosome amplification/absence of centrosomes and CNI and he/she solicits an explanation.

This reviewer asks how can high CNI tumors have almost no centrosome amplification. A good example is #TT44, shown in Figure 2A, field 2. In this tumor, the frequency of centrosome amplification is low, but one can see that there is a high number of centrosomes in a few cells- actually in a very small number of cells. Although we did not give details, some of these cells can have over 40 centrosomes and so this will greatly contribute to increase the CNI. We should emphasize here that the frequency of centrosome amplification was calculated as any event that has more than two centrosomes. Therefore, one event with 3 centrosomes or with 30 will contribute to this frequency in a similar manner. Thus, the differences between high CNI and frequency of centrosome amplification can in part be explained by this (please see below).

Considering the second point, why certain tumors with low CNI have only a small percentage of cells without centrosomes. A possible explanation, that may account also for the lack of correlation between CNI and the frequency of cells with centrosome amplification and centrosome absence is the remaining population of cells. The ones with 1 or 2 centrosomes. If the frequency of cells with one centrosome is quite high this will certainly contribute to a lower CNI.

We mentioned in the revised version- page 9- " We focused on tissue regions where the presence/absence of centrosomes could be easily distinguished". This is

certainly a drawback when identifying nuclei without centrosomes. Indeed, if there is a centrosome positioned between two nuclei, we cannot ascertain if it belongs to one nucleus or the other and so these were not taken into consideration. Moreover, in groups of nuclei that are arranged in cyst-like manner with the centrosomes positioned towards a lumen, it is very difficult to know which centrosome belongs to each nucleus. In this type of arrangements, we just do not take into consideration the nuclei without centrosomes. It is not possible to know if two nuclei and two centrosomes next to it represent, twice one centrosome- one nucleus; 2centrosomes-one nucleus and zero centrosomes-one nucleus. Therefore, we think that the frequency of absence of centrosomes may even be an underestimation. This is why, we argue that the CNI is the most adequate way of characterizing these tumors.
We realize that we have not really explain this point correctly in the revised version of the manuscript. We have now included more explanations (in green) in the results and discussion sections. Further, we have also provided more images to illustrate differences between CNI and the frequencies shown in Figure 3C and D. These are now shown in the new Figure 3A.

Referee #2 (Comments on Novelty/Model System for Author):

This work analyzes centrosome numbers in primary tumors. Something that has rarely been done.

Referee #2 (Remarks for Author):

The authors have clearly made an effort to incorporate reviewer comments into the revised manuscript. I consider the characterization of centrosome numbers in patient tumour samples a critical step for the field that fills a significant gap.

We thank the reviewer for recognizing our efforts and the importance of this work in providing the analysis of patient samples.

My major problem with the original version of this paper was the use of the CNI metric. Unfortunately, the additional data presented in the revised version of this manuscript has only reinforced my initial concern. Fig 3 is especially problematic.

The CNI and the frequency of centrosome amplification do not take into consideration the same parameters. The CNI is calculated by counting all the nuclei and all the centrosomes present in a given tumor section. The frequency of centrosome amplification takes into consideration just the event of having more than 2 centrosomes. Considering the frequency of the absence of centrosomes, this is more difficult to determine than centrosome amplification. Within a tumor section, there are regions with nuclei without centrosomes and these are easy to distinguish. However, in certain (and frequent situations), centrosomes are positioned away from several nuclei and so it is not possible to know which nucleus does not contain a centrosome (the CNI will be below 1, as there are more nuclei than centrosomes).

We decided to not take into account these situations, as they are difficult to evaluate, when calculating the frequency of nuclei without centrosomes. Therefore, the frequencies of no centrosome are most likely underestimated. We will explain this in the results, methods and discussion. We also provided new figures illustrating all these points (new Figure 3A).

Consider the tumour with the highest CNI: TT21. This tumour shows almost no centrosome amplification and has a 3% frequency of nuclei without centrosomes. Thus, I cannot understand how a tumour can have a high CNI without centrosome amplification? CHEMOTHERAPY

We thank this reviewer for bringing this subject up. We actually realized that there was an error in the plot representing the frequency of centrosome amplification. We reviewed all the data again. There is no major difference in terms of the overall frequency of centrosome amplification, but actually in the tumor #TT21, the frequency of centrosome amplification reached 1.5%. We have verified all the others and corrected this one.

Still, as brough up by this reviewer, there are other high CNI tumors with low centrosome amplification frequency. The CNI of a tumor is calculated based on all the centrosomes and all the nuclei of every tumor section. Even with low centrosome amplification, if most neighboring nuclei have two centrosomes, this will account to increase the CNI. In contrast, if there are many nuclei with only one centrosome the CNI will be lower. Please refer to new Figure 3 and the new text section for more explanations.

Conversely, the tumour with the lowest CNI is TT61. The tumour shows almost no centrosome amplification, and only 14% of nuclei do not have centrosomes. Given that the CNI of this tumour is ~0.6, shouldn't more than 14% of cells demonstrate centrosome loss to approach a CNI of about half? Mathematically this doesn't add up. While these two examples are of the tumours with the lowest and highest CNI, all tumours have a poor correlation of CNI with centrosome gain or loss.

We understand the reviewer's comments, but actually we think they reflect the fact that the CNI takes into consideration all the nuclei and so all the cells of a given section including the cells with one or two centrosomes. Further, the CNI will also take into account all the nuclei (independently of their position) and all the centrosomes.

Still on the CNI and the contribution of cells with one or two centrosomes. Take the example of a tumor section with 1000 nuclei. If half the cells have 2 centrosomes and the other half 1 centrosome, the CNI = 1500/1000=1.50. If 30 nuclei instead of having 1 centrosome have zero, the CNI= 1470/1000= 1.47. But if the number of no centrosomes is the same (30) but only 400 nuclei have 2 centrosomes, the CNI=1270/1000=1.27. This example illustrates how the expected centrosome number (one or two centrosomes) impacts the CNI. In the last two examples, the frequency of no centrosomes would be the same, but not the CNI.

To still address an important point, as explained above, we did not take into consideration "absence of centrosomes" to calculate the frequency of no centrosomes shown in graph 3E, unless the nuclei were isolated without any

centrosome or positioned away from centrosomes. Our quantification represents most likely an underestimation. We have explained and give examples with tumor images (new Figure 3) in this revised version of the manuscript.

The rest of the paper characterizes a subset of tumours based on CNI, and conclusions are drawn between centrosome number and clinical outcome. However, the authors have failed to demonstrate a correlation between CNI and centrosome numbers. Would better correlations occur if the authors analyzed tumours with the highest and lowest frequency of centrosome amplification/loss?

One of the major strengths of our work is the number of tumors analyzed and evaluated in a thorough manner. We feel that reducing the number of samples will be impacting in a negative way our study. As explained above, the lack of correlation shows that the CNI of a tumor is multifactorial. In our opinion, there is no other alternative to the CNI in tumors of this type. The CNI will take into consideration, the number of cells without centrosomes, the number of cells with centrosome amplification (and the extent of amplification), and the number of cells that contain one or two centrosomes. As mentioned before, the variability and highly disorganized nature of these tissues do not allow us to determine the exact number of centrosomes in a cell.
We think that we failed to explain this in a straightforward manner and we have included more text and figures to illustrate this point.

This is an important study analyzing centrosome numbers in vivo. I understand it is extremely challenging, and tumour samples are highly heterogeneous, but the current data analysis is insufficient to draw conclusions about centrosome numbers in ovarian tumour samples.

We thank once more this reviewer for recognizing the effort made here. We would like to draw the attention to the fact that the work presented in this article was initiated in 2013. We have attempted in many different ways to generate a robust and accurate view of centrosome numbers in a highly heterogeneous type of tumor. We are extremely confident that our analysis allows us to draw the conclusions exposed here. When we determined the frequency of centrosome amplification and centrosome absence, we even confirmed the CNI of a large group of tumors. We understand that our study reveals unexpected results and raises many new questions. But this is important to advance the fields of centrosome and cancer biology. We propose here that the CNI allows us to compare tumors across our cohort and to obtain data related with genomic and clinical parameters, while the frequencies of centrosome amplification and loss can only be used to infer if a given tumor has more centrosome amplification than loss, for example.

We truly hope that the addition of text and the new figures, the reviewer will agree that the CNI is a measure that translates the centrosome status of these tumors and that it may be used in other heterogeneous tumors.

24th Jun 2022

Dear Dr. Basto,

Thank you for the submission of your revised manuscript to EMBO Molecular Medicine. We have now received feedback from the two reviewers who agreed to re-evaluate your manuscript. As you will see from the reports below, the referees acknowledge the interest of the study but again raise serious and partially overlapping concerns regarding the use of CNI metric.

Taking this in consideration it is clear that publication of the paper cannot be considered at this stage. After our cross-commenting session both referees agreed that a shorter report excluding the CNI metric and including important observations in figures 1, 2, and 5 would be a great contribution to the field and suitable for publication in EMBO Molecular Medicine. If you decide to follow this path, I would like you to consider suggestions of the referee #2 regarding the title and main conclusions outlined in 3 points. Furthermore, I would like you to consider publishing your manuscript as a scientific report (3 figures, ~22000 characters), for more information please check our "Author Guidelines".
https://www.embopress.org/page/journal/17574684/authorguide#reportsarticleguide

Please note that your revised manuscript will not be re-reviewed and will undergo editorial evaluation only. Should you find that the requested revisions are not feasible within the constraints outlined here and choose, therefore, to submit your paper elsewhere, we would welcome a message to this effect.

Yours sincerely,

Zeljko Durdevic

***** Reviewer's comments *****

Referee #1 (Remarks for Author):

I thank the authors for clarifying the points raised. The additional text added in green also helps understanding the meaning of the CNI and its limitations/drawbacks, which was important.

One of the issues raised was the lack of correlation between CNI and centrosome numbers. From the authors explanation, it seems that this could be explained (for centrosome amplification) by the fact that few cells with dozens of centrosomes could give CNI>2 while the overall percentage/frequency of centrosome amplification could still be very low (can even be less than 2 % of the total population which most scientists would consider no amplification). I can understand this math, but this immediately implies that generalisation of the CNI to assess centrosome numbers can be misinterpreted and avoiding this should be prioritised.

It is clear from this work that the patient samples analyzed have almost no centrosome amplification. This may be surprising but it is convincingly demonstrated in my opinion. Thus, shouldn't the authors have focused on low CNI or loss of centrosomes for the analyses? For example, I am not convinced that he role of centrosome amplification can be inferred in tumours that barely contain amplified centrosomes. I would suggest the authors to change or remove sentences such as this one on page 6 : "Surprisingly, we found that the frequency of centrosome amplification was less important than could be predicted from the literature mostly based on cell culture". I do not think this is what this work suggests or assesses.

Also, on page 9 that authors write: "only 9% of tumors exhibited centrosome amplification with a CNI above 2, when defined by the presence of more than 2 centrosomes per cell". I was confused by this, is CNI being used to determine centrosome number? Because from the authors explanation of what the CNI is and from the data provided this is not straightforward and due to variability it is impossible to infer frequency of centrosome amplification based on this. Not sure if I understood this sentence correctly but for me it reads as CNI>2 is equivalent to more than 2 centrosomes per cell? Which cannot be inferred.

Perhaps the use the CNI is more appropriate as 'biomarker' without trying to infer numbers of centrosomes, which I think is important to interpret the data presented here.

Referee #2 (Comments on Novelty/Model System for Author):

See below. The CNI index has major limitations and should be abandoned.

Referee #2 (Remarks for Author):

I appreciate the authors taking the time to address my comments; however, my concerns about CNI are further strengthened. It's clear from the data presented in Figure 3 that CNI is not a useful metric, and it should not be used to characterize numerical centrosome aberrations in cancer. The revised manuscript illustrates the problem of the method used to derive the CNI. For instance, a small number of cells with massive centrosome amplification will drive a big shift in the CNI index and may cancel out the numerical impact of having many cells in the tumor that have lost centrosomes. In such cases, the CNI index may be 'normal', yet extensive aberrations in centrosome numbers could be present. The centrosome number in tumor samples is extremely heterogenous and the averaging used to calculate CNI diminishes any predictive power of the CNI. Based on the evidence presented in the images, I don't see how the CNI could predict tumor biology. In addition, the selection of ~1.45 centrosomes per cell as the cutoff to define tumors with a high or low CNI does not make logical sense within the context of centrosome biology. >2 centrosomes per cell are considered amplification, and <1 centrosome per cell is considered centrosome loss. Using ~1.45 centrosomes per cell as a cutoff highlights the problem of averaging centrosome defects and losing the useful information in the process. I appreciate that calculating the frequency of centrosome loss and gain is not possible with the current staining methods. However, this does not make the CNI a helpful metric.

I understand that this manuscript was an enormous amount of work and attempts to examine centrosome numbers in patient samples, which has rarely been done. However, the current version of the manuscript lists caveats after every conclusion, and I cannot support the publication of clinical correlations derived from the CNI. The data in Figures 3 C and D demonstrate that CNI doesn't adequately represent the alterations in centrosome number in tumors. I would like to propose an option for transforming the data into a rigorous manuscript that will be of value to the field. One option to build off the strengths of this work would be an abbreviated report of the manuscript that would include figures 1, 2, and 5. The centrosome staining in patient tumor samples is nice and demonstrates the heterogeneity of centrosome number among individual cells within a tumor sample and the challenge of quantifying this. This is particularly striking in Fig 3A. Perhaps a shorter version of this manuscript could include more of these representative images from patient tumors. In addition, the field would benefit from a discussion on how the authors tried to quantitate centrosome numbers and the technical challenges of this task. The lack of a cell outline marker linking centrosomes

to cells and the use of a metric that averages across an entire sample are limitations that will need to be overcome in the future. Moreover, the title could be changed to something more descriptive such as: A catalog of numerical centrosome aberrations in ovarian cancer. As I see it, the three main conclusions that can be drawn from this work are 1) Rigorous quantitation of centrosome number in tumor samples is extremely challenging due to tumor heterogeneity, cell disorganization, and technical staining limitations; and 2) Centrosome gains are not as frequent as we might expect from the existing literature in mouse, drosophila, and cell culture cancer models. 3) Centrosome loss events can also be clearly detected in tumors. I believe that with these changes, the manuscript will provide useful information for the centrosome and cancer field at large and avenues for improvement in the future.

Response to reviewers

Referee #1 (Remarks for Author):

I thank the authors for clarifying the points raised. The additional text added in green also helps understanding the meaning of the CNI and its limitations/drawbacks, which was important.

One of the issues raised was the lack of correlation between CNI and centrosome numbers. From the authors explanation, it seems that this could be explained (for centrosome amplification) by the fact that few cells with dozens of centrosomes could give CNI>2 while the overall percentage/frequency of centrosome amplification could still be very low (can even be less than 2 % of the total population which most scientists would consider no amplification). I can understand this math, but this immediately implies that generalisation of the CNI to assess centrosome numbers can be misinterpreted and avoiding this should be prioritised.

It is clear from this work that the patient samples analyzed have almost no centrosome amplification. This may be surprising but it is convincingly demonstrated in my opinion. Thus, shouldn't the authors have focused on low CNI or loss of centrosomes for the analyses? For example, I am not convinced that he role of centrosome amplification can be inferred in tumours that barely contain amplified centrosomes. I would suggest the authors to change or remove sentences such as this one on page 6 : "Surprisingly, we found that the frequency of centrosome amplification was less important than could be predicted from the literature mostly based on cell culture". I do not think this is what this work suggests or assesses.

Also, on page 9 that authors write: "only 9% of tumors exhibited centrosome amplification with a CNI above 2, when defined by the presence of more than 2 centrosomes per cell". I was confused by this, is CNI being used to determine centrosome number? Because from the authors explanation of what the CNI is and from the data provided this is not straightforward and due to variability it is impossible to infer frequency of centrosome amplification based on this. Not sure if I understood this sentence correctly but for me it reads as CNI>2 is equivalent to more than 2 centrosomes per cell? Which cannot be inferred.

Perhaps the use the CNI is more appropriate as 'biomarker' without trying to infer numbers of centrosomes, which I think is important to interpret the data presented here.

We are happy to see that this reviewer considers our explanation of the drawbacks of the CNI. This reviewer is convinced of the frequency of low CNI in EOCs, even if he does not believe that the CNI is useful tool to characterize tumors in situ. As this was also the case for reviewer #2, we have altered the text. We have focus this work on the description of the multiple scenarios of centrosomes in EOCs and how they do not correlate with increased migration or invasion.

The reviewer suggests that we should have focused on the effects of the loss of centrosomes. This is what we have done, in former Figure 5 and EV4-5, when we

characterized the consequences of centrinone treatment in migration and invasion assays of ovarian cancer cell lines. This will now be presented in Figure 3 and EV1.

Considering certain points like the one referred to on page 9, since all information related with the CNI has been removed, this section is no longer present. The sentence related with centrosome amplification in tumors and cell lines was removed as requested by this reviewer.

Referee #2 (Comments on Novelty/Model System for Author):

See below. The CNI index has major limitations and should be abandoned.

Referee #2 (Remarks for Author):

I appreciate the authors taking the time to address my comments; however, my concerns about CNI are further strengthened. It's clear from the data presented in Figure 3 that CNI is not a useful metric, and it should not be used to characterize numerical centrosome aberrations in cancer. The revised manuscript illustrates the problem of the method used to derive the CNI. For instance, a small number of cells with massive centrosome amplification will drive a big shift in the CNI index and may cancel out the numerical impact of having many cells in the tumor that have lost centrosomes. In such cases, the CNI index may be 'normal', yet extensive aberrations in centrosome numbers could be present. The centrosome number in tumor samples is extremely heterogenous and the averaging used to calculate CNI diminishes any predictive power of the CNI. Based on the evidence presented in the images, I don't see how the CNI could predict tumor biology. In addition, the selection of ~1.45 centrosomes per cell as the cutoff to define tumors with a high or low CNI does not make logical sense within the context of centrosome biology. >2 centrosomes per cell are considered amplification, and <1 centrosome per cell is considered centrosome loss. Using ~1.45 centrosomes per cell as a cutoff highlights the problem of averaging centrosome defects and losing the useful information in the process. I appreciate that calculating the frequency of centrosome loss and gain is not possible with the current staining methods. However, this does not make the CNI a helpful metric.

We are truly sorry that after all this work and efforts, this reviewer does not consider the CNI is a valid metric parameter to characterize centrosome number alterations in EOCs.

We certainly understand that it is difficult to consider centrosome numbers as numbers different than 0, 1, 2 or more than 2. But what we tried to convey as a message is that this could be used as a tool to compare centrosome number deviations within a large population of tumors which then could be useful as a tool to consider other parameters. We actually never defined a “normal” CNI for a tumor, as the heterogeneity in centrosome number does not allow to find any “normal” configuration. We proposed the CNI, as this allow us to use sophisticated tools to dichotomize the population and to find correlations that may be exploited in the future. Nevertheless, since both reviewers disagree with the use of the CNI as a metric to characterize centrosome numbers in EOCs, we have taken this reviewer suggestion and removed this information, while maintaining the information related with the frequency of centrosome amplification and centrosome loss.

I understand that this manuscript was an enormous amount of work and attempts to examine centrosome numbers in patient samples, which has rarely been done. However, the current version of the manuscript lists caveats after every conclusion, and I cannot support the publication of clinical correlations derived from the CNI. The data in Figures 3 C and D demonstrate that CNI doesn't adequately represent the alterations in centrosome number in tumors. I would like to propose an option for transforming the data into a rigorous manuscript that will be of value to the field. One option to build off the strengths of this work would be an abbreviated report of the manuscript that would include figures 1, 2, and 5. The centrosome staining in patient tumor samples is nice and demonstrates the heterogeneity of centrosome number among individual cells within a tumor sample and the challenge of quantifying this. This is particularly striking in Fig 3A. Perhaps a shorter version of this manuscript could include more of these representative images from patient tumors. In addition, the field would benefit from a discussion on how the authors tried to quantitate centrosome numbers and the technical challenges of this task. The lack of a cell outline marker linking centrosomes to cells and the use of a metric that averages across an entire sample are limitations that will need to be overcome in the future. Moreover, the title could be changed to something more descriptive such as: A catalog of numerical centrosome aberrations in ovarian cancer. As I see it, the three main conclusions that can be drawn from this work are 1) Rigorous quantitation of centrosome number in tumor samples is extremely challenging due to tumor heterogeneity, cell disorganization, and technical staining limitations; and 2) Centrosome gains are not as frequent as we might expect from the existing literature in mouse, drosophila, and cell culture cancer models. 3) Centrosome loss events can also be clearly detected in tumors. I believe that with these changes, the manuscript will provide useful information for the centrosome and cancer field at large and avenues for improvement in the future.

As suggested by this reviewer, we have shorten this article. It now includes the tumor characterization, in Figure 1-2 and the assays related with mesothelial clearance and invasion through the basement membrane (Figure 3 and EV1). We have also taken up on the suggestion of the title and of all other points. We have removed the sentence related with the comparison with cancer cells in culture since this was requested by reviewer#1. Related with flies and mouse, we decided not to add this comparison,

because in both flies and mice, the studies have been done using PLK4 over-expression as a mean to induce centrosome amplification.

We thank the reviewer or acknowledging the amount of work and human contributors to this article.

15th Jul 2022

Dear Dr. Basto,

Thank you for the submission of your revised manuscript to EMBO Molecular Medicine. I am pleased to inform you that we will be able to accept your manuscript pending the following final amendments:

1) In the main manuscript file, please do the following:
- In M&M, include a statement that the experiments using human samples conformed to the principles set out in the WMA Declaration of Helsinki and the Department of Health and Human Services Belmont Report.
- In the figure legends and EV figure legends where appropriate please indicate the number and nature of replicates and define the error bars (e.g. mean {plus minus} SD).
- Provide data availability statement. If no data are deposited in public repositories, please add the sentence: "This study includes no data deposited in external repositories".

Please check "Author Guidelines" for more information.
https://www.embopress.org/page/journal/17574684/authorguide#availabilityofpublishedmaterial
- Add EV figure legends to the main manuscript file.
2) EV Content: Please upload table from EV Figure 1 as a separate EV Table file (EV Table 1) and update the callouts in the text.
3) Source data: Please zipp source data for EV Figures as one file.
4) Synopsis:
- Please provide visual abstract as a high-resolution jpeg file 550 px-wide x (250-400)-px high.
- Please check your synopsis text and image and submit their final versions with your revised manuscript. Please be aware that in the proof stage minor corrections only are allowed (e.g., typos).
5) For more information: There is space at the end of each article to list relevant web links for further consultation by our readers. Could you identify some relevant ones and provide such information as well? Some examples are patient associations, relevant databases, OMIM/proteins/genes links, author's websites, etc...
6) As part of the EMBO Publications transparent editorial process initiative (see our Editorial at http://embomolmed.embopress.org/content/2/9/329), EMBO Molecular Medicine will publish online a Review Process File (RPF) to accompany accepted manuscripts. This file will be published in conjunction with your paper and will include the anonymous referee reports, your point-by-point response and all pertinent correspondence relating to the manuscript. Let us know whether you agree with the publication of the RPF and as here, if you want to remove or not any figures from it prior to publication. Please note that the Authors checklist will be published at the end of the RPF.
7) Please provide a point-by-point letter to my comments and to the reviewer's reports with your detailed responses (as separate Word files).

I look forward to reading a new revised version of your manuscript as soon as possible.

Yours sincerely,

Zeljko Durdevic

The authors performed the requested editorial changes.

We are pleased to inform you that your manuscript is accepted for publication and is now being sent to our publisher to be included in the next available issue of EMBO Molecular Medicine.

EMBO Press Author Checklist

Corresponding Author Name:	
Journal Submitted to:	
Manuscript Number:	

USEFUL LINKS FOR COMPLETING THIS FORM
The EMBO Journal - Author Guidelines
EMBO Reports - Author Guidelines
Molecular Systems Biology - Author Guidelines
EMBO Molecular Medicine - Author Guidelines

Reporting Checklist for Life Science Articles (updated January 2022)
This checklist is adapted from Materials Design Analysis Reporting (MDAR) Checklist for Authors. MDAR establishes a minimum set of requirements in transparent reporting in the life sciences (see Statement of Task: 10.31222/osf.io/9sm4x). Please follow the journal's guidelines in preparing your manuscript.
Please note that a copy of this checklist will be published alongside your article.

Abridged guidelines for figures
1. Data
The data shown in figures should satisfy the following conditions:
- the data were obtained and processed according to the field's best practice and are presented to reflect the results of the experiments in an accurate and unbiased manner.
- ideally, figure panels should include only measurements that are directly comparable to each other and obtained with the same assay.
- plots include clearly labeled error bars for independent experiments and sample sizes. Unless justified, error bars should not be shown for technical replicates.
- if n<5, the individual data points from each experiment should be plotted. Any statistical test employed should be justified.
- Source Data should be included to report the data underlying figures according to the guidelines set out in the authorship guidelines on Data Presentation.

2. Captions
Each figure caption should contain the following information, for each panel where they are relevant:
- a specification of the experimental system investigated (eg cell line, species name).
- the assay(s) and method(s) used to carry out the reported observations and measurements.
- an explicit mention of the biological and chemical entity(ies) that are being measured.
- an explicit mention of the biological and chemical entity(ies) that are altered/varied/perturbed in a controlled manner.
- the exact sample size (n) for each experimental group/condition, given as a number, not a range;
- a description of the sample collection allowing the reader to understand whether the samples represent technical or biological replicates (including how many animals, litters, cultures, etc.).
- a statement of how many times the experiment shown was independently replicated in the laboratory.
- definitions of statistical methods and measures:
  - common tests, such as t-test (please specify whether paired vs. unpaired), simple χ2 tests, Wilcoxon and Mann-Whitney tests, can be unambiguously identified by name only, but more complex techniques should be described in the methods section;
  - are tests one-sided or two-sided?
  - are there adjustments for multiple comparisons?
  - exact statistical test results, e.g., P values = x but not P values < x;
  - definition of 'center values' as median or average;
  - definition of error bars as s.d. or s.e.m.

Please complete ALL of the questions below.
Select "Not Applicable" only when the requested information is not relevant for your study.

Materials

Newly Created Materials	Information included in the manuscript?	In which section is the information available? (Reagents and Tools Table, Materials and Methods, Figures, Data Availability Section)
New materials and reagents need to be available; do any restrictions apply?	Yes	Materials and Method

Antibodies	Information included in the manuscript?	In which section is the information available? (Reagents and Tools Table, Materials and Methods, Figures, Data Availability Section)
For **antibodies** provide the following information:<br>- Commercial antibodies: RRID (if possible) or supplier name, catalogue number and or/clone number<br>- Non-commercial: RRID or citation	Yes	Materials and Methods

DNA and RNA sequences	Information included in the manuscript?	In which section is the information available? (Reagents and Tools Table, Materials and Methods, Figures, Data Availability Section)
**Short novel DNA or RNA including primers, probes**: provide the sequences.	Not Applicable	

Cell materials	Information included in the manuscript?	In which section is the information available? (Reagents and Tools Table, Materials and Methods, Figures, Data Availability Section)
**Cell lines:** Provide species information, strain. Provide accession number in repository **OR** supplier name, catalog number, clone number, and/**OR** RRID.	Yes	Materials and Method
**Primary cultures:** Provide species, strain, sex of origin, genetic modification status.	Not Applicable	
Report if the cell lines were recently **authenticated** (e.g., by STR profiling) and tested for mycoplasma contamination.	Yes	Materials and Method

Experimental animals	Information included in the manuscript?	In which section is the information available? (Reagents and Tools Table, Materials and Methods, Figures, Data Availability Section)
**Laboratory animals or Model organisms:** Provide species, strain, sex, age, genetic modification status. Provide accession number in repository **OR** supplier name, catalog number, clone number, **OR** RRID.	Yes	Materials and Method
**Animal observed in or captured from the field:** Provide species, sex, and age where possible.	Not Applicable	
Please detail **housing and husbandry conditions**.	Yes	Materials and Method

Plants and microbes	Information included in the manuscript?	In which section is the information available? (Reagents and Tools Table, Materials and Methods, Figures, Data Availability Section)
**Plants:** provide species and strain, ecotype and cultivar where relevant, unique accession number if available, and source (including location for collected wild specimens).	Not Applicable	
**Microbes:** provide species and strain, unique accession number if available, and source.	Not Applicable	

Human research participants	Information included in the manuscript?	In which section is the information available? (Reagents and Tools Table, Materials and Methods, Figures, Data Availability Section)
If collected and within the bounds of privacy constraints report on age, sex and gender or ethnicity for all study participants.	Not Applicable	

Core facilities	Information included in the manuscript?	In which section is the information available? (Reagents and Tools Table, Materials and Methods, Figures, Data Availability Section)
If your work benefited from core facilities, was their service mentioned in the acknowledgments section?	Yes	Acknowledgments

Design

Study protocol	Information included in the manuscript?	In which section is the information available? (Reagents and Tools Table, Materials and Methods, Figures, Data Availability Section)
If study protocol has been **pre-registered, provide DOI in the manuscript**. For clinical trials, provide the trial registration number **OR** cite DOI.	Not Applicable	
Report the **clinical trial registration number** (at ClinicalTrials.gov or equivalent), where applicable.	Not Applicable	

Laboratory protocol	Information included in the manuscript?	In which section is the information available? (Reagents and Tools Table, Materials and Methods, Figures, Data Availability Section)
Provide DOI OR other citation details if **external detailed step-by-step protocols** are available.	Not Applicable	

Experimental study design and statistics	Information included in the manuscript?	In which section is the information available? (Reagents and Tools Table, Materials and Methods, Figures, Data Availability Section)
Include a statement about **sample size** estimate even if no statistical methods were used.	Yes	Materials and Methods, Figures, Figures legend
Were any steps taken to minimize the effects of subjective bias when allocating animals/samples to treatment (e.g. **randomization procedure**)? If yes, have they been described?	Not Applicable	
Include a statement about **blinding** even if no blinding was done.	Yes	Material and methods
Describe **inclusion/exclusion criteria** if samples or animals were excluded from the analysis. Were the criteria pre-established?<br><br>If sample or data points were omitted from analysis, report if this was due to attrition or intentional exclusion and provide justification.	Yes	Material and Methods
For every figure, are **statistical tests** justified as appropriate? Do the data meet the assumptions of the tests (e.g., normal distribution)? Describe any methods used to assess it. Is there an estimate of variation within each group of data? Is the variance similar between the groups that are being statistically compared?	Yes	figures, main text, Data source

Sample definition and in-laboratory replication	Information included in the manuscript?	In which section is the information available? (Reagents and Tools Table, Materials and Methods, Figures, Data Availability Section)
In the figure legends: state number of times the experiment was **replicated** in laboratory.	Yes	Figures legend
In the figure legends: define whether data describe **technical or biological replicates**.	Yes	Figures legend

Ethics

Ethics	Information included in the manuscript?	In which section is the information available? (Reagents and Tools Table, Materials and Methods, Figures, Data Availability Section)
Studies involving **human participants**: State details of **authority granting ethics approval** (IRB or equivalent committee(s), provide reference number for approval.	Yes	Materials and Methods
Studies involving **human participants**: Include a statement confirming that **informed consent** was obtained from all subjects and that the experiments conformed to the principles set out in the WMA Declaration of Helsinki and the Department of Health and Human Services Belmont Report.	Yes	Materials and Methods
Studies involving **human participants:** For publication of **patient photos**, include a statement confirming that consent to publish was obtained.	Not Applicable	
Studies involving experimental **animals**: State details of **authority granting ethics approval** (IRB or equivalent committee(s), provide reference number for approval. Include a statement of compliance with ethical regulations.	Not Applicable	
Studies involving **specimen and field samples:** State if relevant **permits** obtained, provide details of authority approving study; if none were required, explain why.	Yes	Materials and Methods

Dual Use Research of Concern (DURC)	Information included in the manuscript?	In which section is the information available? (Reagents and Tools Table, Materials and Methods, Figures, Data Availability Section)
Could your study fall under dual use research restrictions? Please check biosecurity documents and list of **select agents and toxins** (CDC): https://www.selectagents.gov/sat/list.htm	Not Applicable	
If you used a select agent, is the security level of the lab appropriate and reported in the manuscript?	Not Applicable	
If a study is subject to dual use research of concern regulations, is the name of the **authority granting approval and reference number** for the regulatory approval provided in the manuscript?	Not Applicable	

Reporting

The MDAR framework recommends adoption of discipline-specific guidelines, established and endorsed through community initiatives. Journals have their own policy about requiring specific guidelines and recommendations to complement MDAR.

Adherence to community standards	Information included in the manuscript?	In which section is the information available? (Reagents and Tools Table, Materials and Methods, Figures, Data Availability Section)
State if relevant guidelines or checklists (e.g., **ICMJE, MIBBI, ARRIVE, PRISMA**) have been followed or provided.	Yes	Checklist BRISQ was followed
For **tumor marker prognostic studies**, we recommend that you follow the **REMARK** reporting guidelines (see link list at top right). See author guidelines, under 'Reporting Guidelines'. Please confirm you have followed these guidelines.	Not Applicable	
For **phase II and III randomized controlled trials**, please refer to the **CONSORT** flow diagram (see link list at top right) and submit the CONSORT checklist (see link list at top right) with your submission. See author guidelines, under 'Reporting Guidelines'. Please confirm you have submitted this list.	Not Applicable	

Data Availability

Data availability	Information included in the manuscript?	In which section is the information available? (Reagents and Tools Table, Materials and Methods, Figures, Data Availability Section)
Have **primary datasets** been deposited according to the journal's guidelines (see 'Data Deposition' section) and the respective accession numbers provided in the Data Availability Section?	Not Applicable	
Were **human clinical and genomic datasets** deposited in a public access-controlled repository in accordance to ethical obligations to the patients and to the applicable consent agreement?	Not Applicable	
Are **computational models** that are central and integral to a study available without restrictions in a machine-readable form? Were the relevant accession numbers or links provided?	Not Applicable	
If publicly available data were reused, provide the respective **data citations in the reference list**.	Not Applicable	
